# WDR72 Enhances the Stemness of Lung Cancer Cells by Activating the AKT/HIF-1*α* Signaling Pathway

**DOI:** 10.1155/2022/5059588

**Published:** 2022-11-07

**Authors:** Xiaoping Ouyang, Xinlin Shi, Na Huang, Yuping Yang, Wei Zhao, Wei Guo, Yumin Huang

**Affiliations:** ^1^Department of Respiratory and Critical Care Medicine, The Affiliated Hospital of Yangzhou University, Yangzhou 225002, China; ^2^Department of Pulmonary and Critical Care Medicine, The First Affiliated Hospital of Chengdu Medical College, Chengdu 610500, China; ^3^School of Laboratory Medicine, Chengdu Medical College, Chengdu 610500, China

## Abstract

**Objectives:**

Lung cancer is a common malignant tumor with high morbidity and mortality rate. Lung cancer stem cells are crucial in the development of lung cancer. In this study, we investigate WD repeat-containing protein 72 (WDR72) on lung cancer cell stemness and explore its underlying mechanism.

**Methods:**

WDR72 expression was investigated in lung cancer tissues and lung cancer stem cells by Western blot and RT-qPCR. The stemness of lung cancer stem cells was verified by the sphere-forming experiment and the abundance of stem cell markers. For the purpose of determining lung cancer stem cell growth, metastasis, and apoptosis, the CCK-8 assay, colony formation, Transwell migration, and flow cytometry were carried out. The ability of tumorigenesis *in vivo* was explored by xenograft tumor mouse models.

**Results:**

Up-regulation of WDR72 was found in lung cancer tissues and lung cancer stem cells. WDR72 overexpression significantly activated the AKT/HIF-1*α* signaling pathway. Application of PI3K/AKT pathway inhibitor LY29004 was able to counteract the impacts of WDR72 upregulation on genes related to stemness, growth, migration, and apoptosis in lung cancer stem cells. The sphere formation of lung cancer stem cells was significantly diminished after inhibiting the AKT/HIF-1*α* pathway. The promotion of WDR72 overexpression on lung cancer stem cell proliferation and metastasis was also eliminated by LY29004 treatment.

**Conclusion:**

WDR72 activates the AKT/HIF-1*α* signaling pathway to enhance the stemness of lung cancer stem cells and promote the growth and metastasis of lung cancer.

## 1. Introduction

Lung cancer has been of great concern worldwide due to its high incidence and high mortality rate [1]. Data released by the World Health Organization in 2020 revealed that lung cancer is the second most prevalent and the deadliest tumor worldwide [2]. Moreover, lung cancer accounts for the highest number of new cancer cases and cancer-related deaths in China in 2020 [3]. For patients with advanced lung cancer, chemotherapy or combination therapy is the main clinical treatment strategy. Although neoadjuvant therapy has improved the quality of life in some patients, drug resistance and recurrence make the clinical treatment of lung cancer difficult, and are the key reasons for the high mortality rate of lung cancer [4].

In recent years, cancer stem cells (CSCs) have become hot spots in cancer research. Since they are first identified in leukemia, cancer stem cells have been isolated from majority of solid tumors such as breast cancer, brain glioma, prostate cancer, hepatocarcinoma, and colorectal cancer, and their activity on tumorigenesis has been demonstrated in several cancer types [5, 6]. Lung cancer stem cells (LCSCs) are of vital importance in the progression of lung cancer. In 2005, researchers identified a population of cells at the junction of bronchial and alveolar ducts that could develop into lung adenocarcinoma after K-RAS mutation [7]. In 2007, a research team isolated a subpopulation of cells with stem-like characteristics from lung cancer tissues and cells, using the high efflux capacity of stem cells for Hoechst 33342 dye, and demonstrated that this group of cells are multidirectional differentiation, self-renewal, reconstituting tumor tissues, and is highly efficient in tumorigenesis *in vivo* [8]. Another study found that lung cancer cells that survived multiple chemotherapeutic agents were highly expressed with stem cell marker proteins and that this group of cells had stemness characteristics such as self-renewal and sphere formation *in vitro* [9]. Accumulating evidence has demonstrated the existence of the small population of stem-like tumor cells in lung cancer is capable of multidirectional differentiation, self-renewal, unlimited proliferation, and efficient tumor formation *in vivo* [10]. The survival of these stem-like cells in targeted therapy, chemotherapy, radiotherapy, and immunotherapy is a leading cause of recurrence and drug resistance in lung cancer [11]. Although CSCs have been widely reported in various types of tumors, their mechanisms in lung cancer are still poorly understood [12]. Thus, there is an urgent need for research on lung cancer stem cells.

WD repeat-containing protein 72 (WDR72), a member of the WD40-repeat domain superfamily [13], is a scaffolding protein with no intrinsic enzymatic activity and forms multiple *β*-propeller blade structures [14]. Previous studies on WDR72 were mainly focused on amelogenesis imperfecta (AI), while its role in malignant tumor was rarely reported [14–16]. The function of WDR72 has only been mentioned in a few cancers. WDR72 is a potential biomarker for predicting the risk of recurrence of bladder cancer [17]. Furthermore, WDR72 could be employed to assist in the diagnosis of esophageal cancer [18]. WDR72 is a tumor suppressor that has the potential to treat renal cell carcinoma, and low expression of WDR72 suggested shortened survival of patients [19].

The PI3K/AKT signaling pathway is a key pathway that influences lung cancer progression. Emerging data showed that the PI3K/AKT/mTOR pathway can be a therapeutic target for NSCLC [20, 21]. The activation of the PI3K/AKT pathway can enhance tumor vascularization and tumor growth [22], while inhibitors of this pathway can significantly inhibit lung cancer development [23, 24]. HIF-1*α* is a transcription factor widely expressed in humans under hypoxic environments and is highly expressed in most tumor tissues [25]. It has been shown that high expression of HIF-1*α* is a main initiator of epithelial-mesenchymal transition in various types of cancer cells [26, 27]. HIF-1 was identified as a downstream regulatory molecule of the PI3K/AKT pathway [28].

However, no correlation between WDR72 and lung cancer was explored before. Therefore, the goal of this study is to investigate the influence of WDR72 on the stemness of lung cancer cells and provide a new treatment strategy for lung cancer.

## 2. Methods

### 2.1. Clinical Samples

All tumor tissues and the paired normal tissues were obtained from 47 patients with clinical diagnoses of lung cancer who underwent surgery in the Department of Respiratory and Critical Care Medicine of The Affiliated Hospital of Yangzhou University from May 2020 to August 2021. The specimen collection has been approved by the ethics committee of Affiliated Hospital of Yangzhou University (1789r33). Written informed consent was obtained from all the patients prior to enrollment in the study.

### 2.2. Cell Culture and Transfection

Lung cancer cells BEAS-2B, A549, SPCA-1, NCI-H270, PC-9, and H1975 were cultured in DMEM, supplemented with 10% FBS. A549 and SPCA-1 stem cells were isolated from A549 and SPCA-1, respectively. In brief, A549 and SPCA-1 cells were seeded on 6-well plates at the concentration of 1 × 10^3^ cells/well in DMEM media supplemented with FBS for 10 days. Then, clones were isolated and cultured in a serum-free medium for another 20 days until spheres formation. Thereafter, the stemness of these stem-like lung cancer cells was assessed by sphere formation assay and the expression of stem cell makers. All the cells were maintained with 5% CO_2_ at 37°C in the incubator. WDR72, sh-WDR72, and vectors were constructed by GeneChem. Cell transfection was carried out by using Lipo 3000 Kit (Invitrogen, USA).

### 2.3. Luciferase Assay

Cignal Finder 10-Pathway Reporter Array (Qiagen) and triple hypoxia response element (3 × HRE) system were employed as our previous reports [29, 30]. Briefly, Cignal Finder 10-Pathway Reporter Array (Qiagen, Shanghai, 336821) was transfected in WDR72 knockdown NSCLC and parental cells following the manufacturer's instructions. 3 × HRE luciferase reporter and transfection efficiency control, renilla luciferase plasmids were cotransfected. After 36 h, the transfected cells were harvested, and luciferase activities were measured by the Dual-Luciferase Reporter Assay System (Promega) according to the manufacturer's protocol.

### 2.4. Xenograft Tumor Mouse Models

The lung cancer cells were prepared by trypsinization and cell suspensions were adjusted to the density of 1 × 10^7^ mL. 4-week-old BALB/c nude mice were provided by Slac Animal (Shanghai, China). Each mouse received a subcutaneous injection of 100 *μ*L of 1 × 10^6^ cells in the hind leg. Tumor size measurement was performed every two days until the average size of the tumors reached 50–100 mm^3^, and the tumors were excised and weighed at the final day. The animal experiment was administrated and approved by the Animal Ethics Committee of Chengdu Medical College.

### 2.5. RT-qPCR

Total RNA of cells was isolated and then cDNA was generated. Quantitative RT-PCR was carried out via the SYBR Green Mix with primers for WDR72, OCT4, CD44, CD133, Cyclin A1, Cyclin B1, E-cadherin, N-cadherin, Bax, and Bcl-2. Ct values were determined by the Applied Biosystems® 7500 PCR Systems.

### 2.6. Sphere Formation Assay

Cells were placed on 6-well plates at the concentration of 1 × 10^3^ cells/well in DMEM media supplemented with 5 mg/L of heparin, 20 *μ*g/L·of hEGF, 2% B27, and 10 *μ*g/L·of bFGF. After 10 days of culture, the tumor spheres were observed and the sphere formation efficiency was calculated.

### 2.7. Western Blot and Immunohistochemistry (IHC)

Cells were lysed in lysis buffer (RIPA + 1%PMSF) for total protein extraction. The proteins were separated on SDS-PAGE electrophoresis and transferred to membranes. The membranes were incubated with primary antibodies at 4°C for 16 hours. After washing with PBST three times, they were hybridized with secondary antibody for 50 minutes at 25°C. Finally, protein bands were detected using the ECL Assay Kit (Applygen, China) and quantified with Image *J*.

Briefly, the tissues were fixed in 4% paraformaldehyde overnight and embedded in paraffin. Then, the sections were stained with the WDR72 antibody, DAB (diaminobenzidine), and hematoxylin. Afterward, they were dehydrated in a gradient alcohol solution. A microscope was used for capturing the stained sections (Carl Zeiss AG, Stuttgart, Germany).

### 2.8. CCK-8 Assay

Cell viability was examined in accordance with the recommended protocol from the CCK-8 kit (MCE, China). About 1 × 10^3^ cells were placed in each well of a 96-well plate. Subsequently, 10 *μ*L of the CCK-8 solution was added to each well. Cells were then cultured for another 3 hours at 37°C. Finally, the OD values of each well were read at 450 nm.

### 2.9. Cell Apoptosis Assay

Cell apoptosis was detected using the Annexin V-FITC kit according to the recommended protocol. In brief, cells were suspended with binding buffer, 5 *μ*l of Annexin V-FITC was added and incubated at 4°C for 10 minutes. Then, 10 *μ*l of PI solution was added and incubated for another 5 minutes at 2–8°C. Finally, cells were analyzed by flow cytometry.

### 2.10. Colony Formation Assay

Cells were placed at a density of 500 cells/well in a 6-well plate, and cultured at 37°C. After 10 days, the supernatant was discarded and the cells were washed. Then, the cells were fixed in paraformaldehyde, and stained with GIMSA. After washing and drying, the number of colonies were counted under a microscope.

### 2.11. Transwell Assay

For migration assay, 1 × 10^5^ cells were suspended and seeded on the upper chamber in a serum-free media. The complete media with 10% serum was placed in the lower chamber. The cells were washed with PBS after 24 hours followed by fixation with 4% paraformaldehyde for 10–20 minutes. Migrated cells were visualized by the microscope.

### 2.12. Statistical Analysis

The experimental data were analyzed using the SPSS software. All the figures were presented as mean ± standard deviation. Comparisons between groups were made using ANOVA or the Student's *t*-test.

## 3. Results

### 3.1. WDR72 is Overexpressed in Lung Cancer Stem Cells

**Figure 1:**
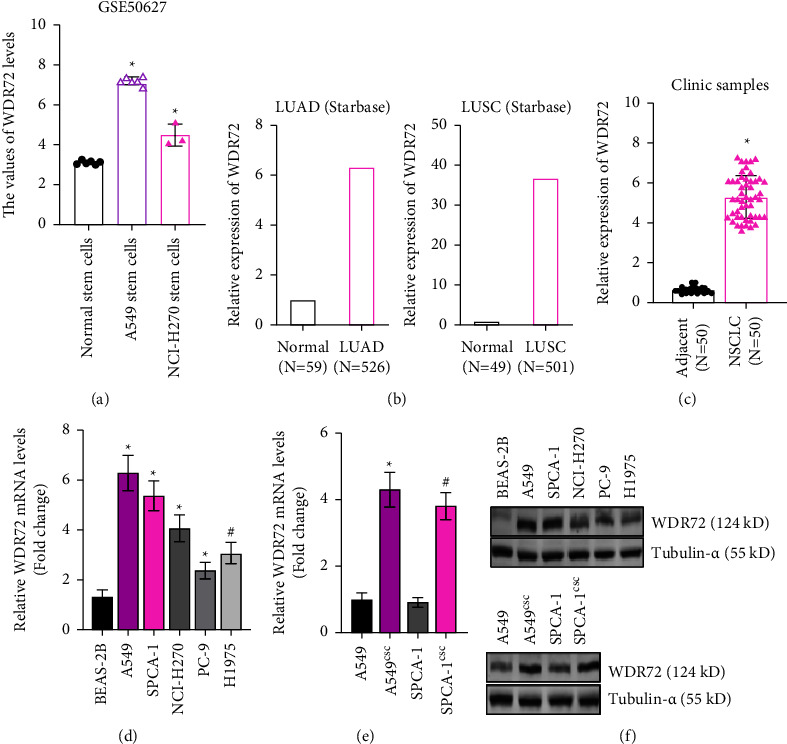
The expression of WDR72 in lung cancer and LCSCs. (a) The expression of WDR72 in LCSCs analyzed in the GEO database. (b) WDR72 level in lung cancer tissues and normal lung tissues analyzed in the Starbase database. (c) RT-qPCR analysis compared the mRNA of WDR72 in NSCLC tissues and normal tissues. (d) The mRNA of WDR72 was examined in normal lung epithelial cells and lung cancer cells. (e) The mRNA level of WDR72 was examined in lung cancer cells and LCSCs. (f) WDR72 protein abundance was examined in normal lung epithelial cells, lung cancer cells, and LCSCs.

To elucidate the role of WDR72 on the development of lung cancer stemness, we detected the expression of WDR72 in lung cancer stem cells (LCSCs). We analyzed data from GEO (https://www.ncbi.nlm.nih.gov/geo/) database, and it was found that A549 stem cells and NCI-H270 stem cells displayed a higher expression of WDR72 (Figure 1(a)). Data collected from the Starbase (https://starbase.sysu.edu.cn) database also demonstrated that lung cancer tissues overexpressed WDR72 (Figure 1(b)). The expression of WDR72 was further examined in lung cancer patients. Results showed that NSCLC tissues expressed more WDR72 than neighboring normal lung tissues (Figure 1(c), Figure S1A). Moreover, patients with high expression of WDR72 exhibited lower survival probability in the public database “Kaplan-Meier Plotter” [31] (Figure S2A). Then the level of WDR72 was detected in normal lung epithelial cell line BEAS-2B, and lung cancer cell lines A549, SPCA-1, NCI-H270, PC-9, and H1975. In comparison to BEAS-2B, WDR72 showed a striking elevation in all the lung cancer cell lines (Figures 1(d) and 1(f)). Subsequently, we compared the WDR72 levels between lung cancer cells (LSCs) and LCSCs which were isolated from LSCs. Both A549 and SPCA-1 stem cells showed higher WDR72 expression (Figures 1(e) and 1(f)). These findings suggested that WDR72 may play a role in lung cancer stemness.

### 3.2. WDR72 Influences the Stemness of LCSCs

**Figure 2:**
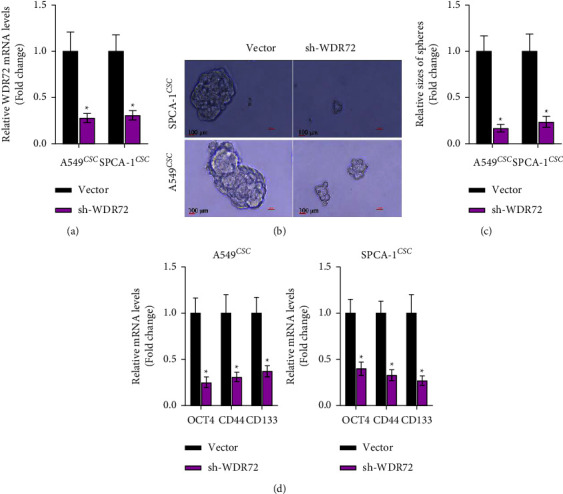
WDR72 knockdown inhibits the stemness of LCSCs. (a) The efficacy of WDR72 knocking down. (b) Evaluation of tumor sphere formation after sh-WDR72 transfection. (c) Statistical data revealed the size of the spheres in the vector group and sh-WDR72 group. (d) RT-qPCR analysis showed the abundance of OCT4, CD44, and CD133 after WDR72 knockdown.

A549 and SPCA-1 stem cells with stable WDR72 knockdown were constructed. WDR72 expression was successfully knocked down in both the A549 and SPCA-1 stem cells, as shown in Figure 2(a). Tumor sphere-forming experiments revealed that cells in the shWDR72 group had considerably weakened sphere-forming ability than those in the empty-vector group (Figures 2(b) and 2(c)). When WDR72 was deregulated in A549 and SPCA-1 stem cells, the mRNA levels of stem cell markers, OCT4, CD44, and CD133, were significantly decreased (Figure 2(d)). These findings suggest that knocking down WDR72 can restrain the expression of stem cell markers and the capacity of LCSCs to form tumor spheres.

### 3.3. WDR72 Knockdown Deters LCSC Growth and Metastasis

**Figure 3:**
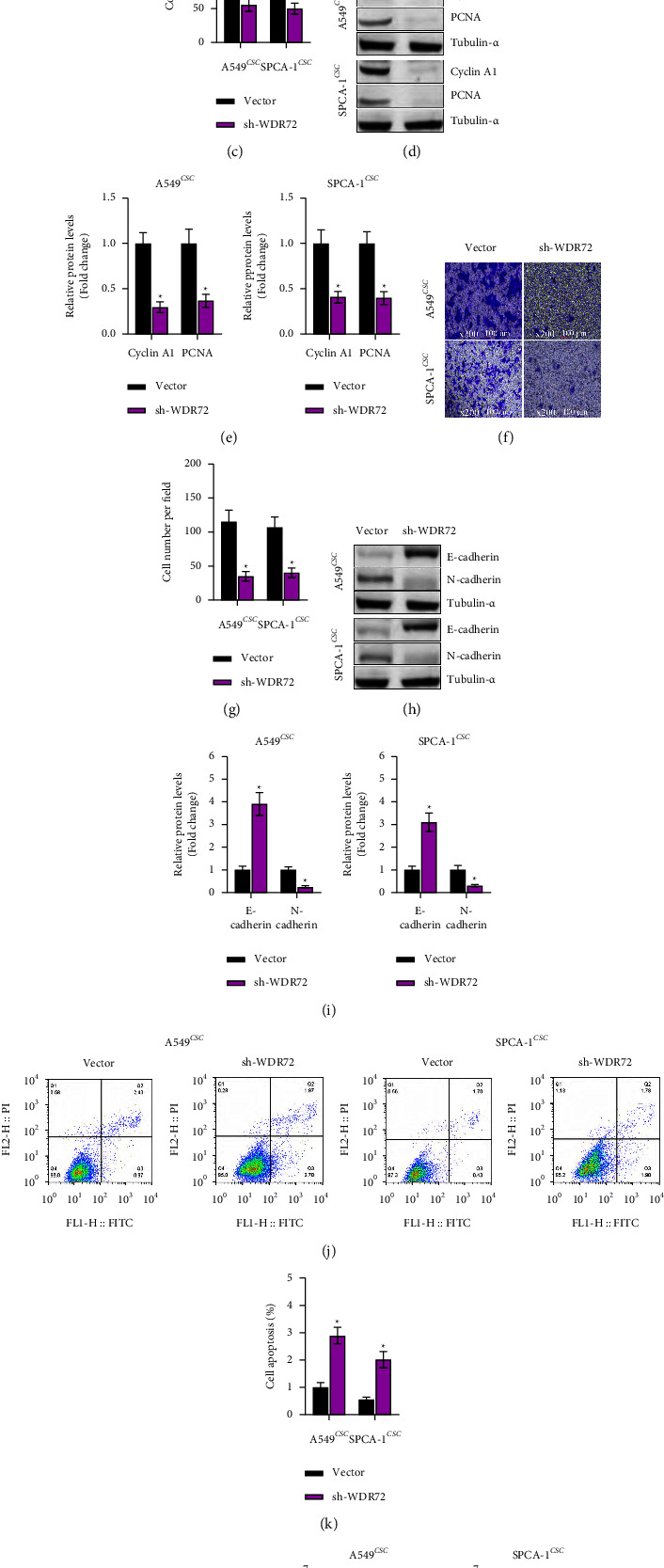
Knockdown of WDR72 inhibits LCSC proliferation and migration, and promotes apoptosis. (a) The viability of LCSCs detected by CCK-8 assay (b) The proliferation of LCSCs detected by colony formation assay. (c) The changes in colony number. (d) and (e) Abundance of proliferation-associated proteins. (f) and (g) The migration of LCSCs detected by Transwell assay. (h) and (i) Abundance of EMT-associated proteins. (j) and (k) The apoptosis of LCSCs analyzed by flow cytometry. (l) and (m) Abundance of apoptosis-associated proteins.

To detect the function of WDR72 knockdown on LCSC growth, we first detected the change of viability of A549 stem cells and SPCA-1 stem cells. CCK-8 assay revealed that knocking down of WDR72 reduced the viability of A549 stem cells and SPCA-1 stem cells at 24 hours, 48 hours, as well as 72 hours (Figure 3(a)). Lung cancer stem cell proliferation was then evaluated by colony formation assay. As shown in Figures 3(b) and 3(c), shWDR72 inhibited lung cancer stem cell proliferation. The abundance of proliferation markers, including cyclin A1 and PCNA, was also reduced by WDR72 downregulation (Figures 3(d) and 3(e)). We next evaluated whether WDR72 knockdown could also inhibit the metastasis of lung cancer stem cells. Transwell assay showed that downregulation of WDR72 strikingly suppressed the migratory capacity of A549 and SPCA-1stem cells (Figures 3(f) and 3(g)). This finding was further confirmed by the Western blot analysis. It was found that the expression of E-cadherin was elevated and N-cadherin inhibited in cells transfected with shWDR72 (Figures 3(h) and 3(i)). To further illustrate the impact of WDR72 knockdown on LCSC growth, we examined the changes in cell apoptosis and found that the shWDR72 group witnessed an elevation of apoptotic cells (Figures 3(j) and 3(k)). Additionally, the abundance of apoptotic activator, Bax, was increased, whereas the expression of apoptotic inhibitor, Bcl-2, was reduced in the shWDR72 cells (Figures 3(l) and 3(m)). The results clued that WDR72 inhibition was responsible for the restraint of lung cancer stem cell growth and metastasis.

### 3.4. Knockdown of WDR72 Weakens the Tumorigenesis of LCSCs *in Vivo*

**Figure 4:**
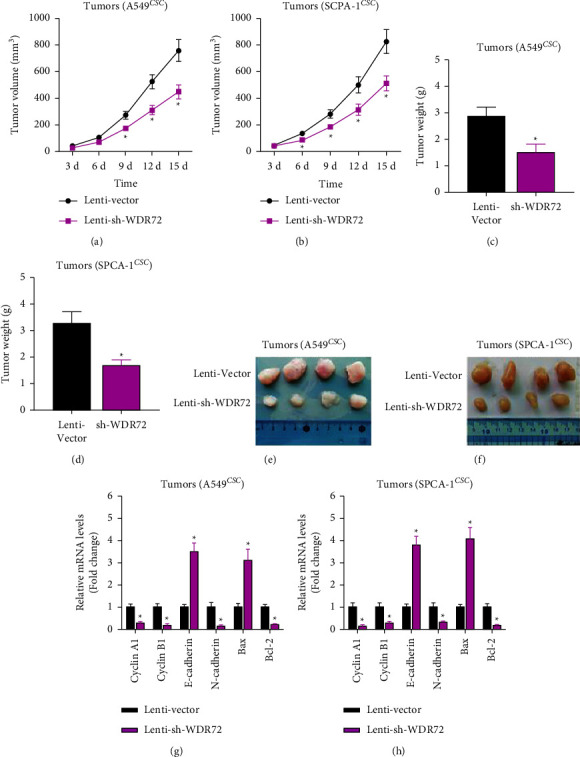
WDR72 knockdown restrains the tumorigenesis of LCSCs. (a) The tumor volume of A549 stem cell-injected mice. (b) The tumor volume of SPCA-1 stem cell-injected mice. (c) The tumor weight of A549 stem cell-injected mice. (d) The tumor weight of SPCA-1 stem cell-injected mice. (e) Tumors excised from A549 stem cell-injected mice. (f) Tumors excised from SPCA-1 stem cell-injected mice. (g) Abundance of proliferation-, EMT-, and apoptosis-associated genes in tumor tissues of A549 stem cell-injected mice. (h) Abundance of proliferation-, EMT-, and apoptosis-associated genes in tumor tissues of SPCA-1 stem cell-injected mice.

A549 and SPCA-1 stem cells were injected subcutaneously into mice to determine the tumorigenic ability of LCSCs. The results showed that the tumor volume in the shWDR72 group was reduced in comparison to that in the vector group (Figures 4(a) and 4(b)). Tumor weight was considerably lower in the shWDR72 group than in the vector group (Figures 4(c) and 4(d)). In addition, knockdown of WDR72 can inhibit tumorigenesis of lung cancer stem cells (Figures 4(e) and 4(f)). To be more specific about the antitumor effect of shWDR72, we measured the abundance of proliferation-, metastasis-, and apoptosis-related markers in tumor tissues. RT-qPCR results showed that the level of proliferation markers, cyclin A1 and cyclin B1, was significantly downregulated in the shWDR72 group (Figures 4(g) and 4(h)). As for EMT markers, the level of E-cadherin was up-regulated and that of N-cadherin was decreased after knocking down of WDR72 (Figures 4(g) and 4(h)). Moreover, the apoptotic activator, Bax, was up-regulated, while the expression of apoptotic inhibitor, Bcl-2, was inhibited in the tumor tissue of the shWDR72 group (Figures 4(g) and 4(h)). These results demonstrated that knockdown of WDR72 inhibited tumorigenesis of LCSCs *in vivo*.

### 3.5. WDR72 Regulates LCSCs through the AKT/HIF-1*α* Signaling Pathway

**Figure 5:**
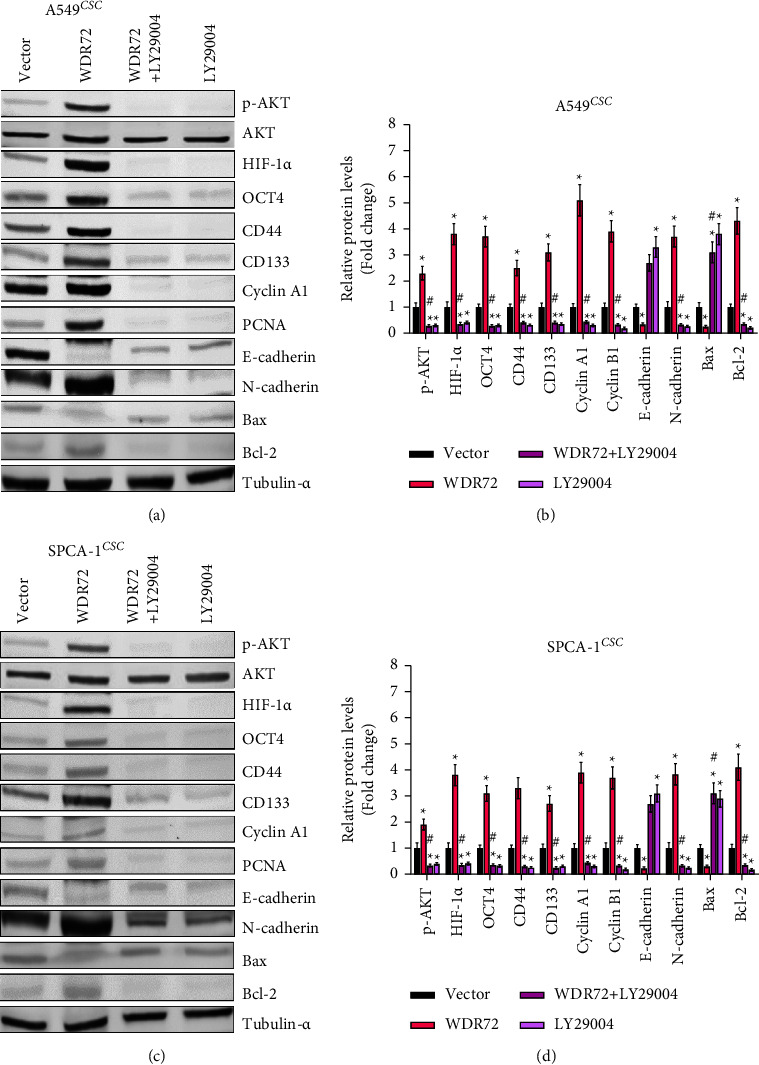
WDR72 regulates lung cancer stem cells via the AKT/HIF-1*α* signaling pathway. (a) and (b) The protein level of stemness, proliferation, metastasis, and apoptosis-related genes in A549 stem cells treated with AKT inhibitor. (c) and (d) The protein level of stemness, proliferation, metastasis, and apoptosis-related genes in SPCA-1 stem cells treated with AKT inhibitors.

To screen the downstream signal pathway(s) of WDR72 in CSC cells, the Cignal Finder Cancer 10-Pathway Reporter Array was employed in cells when knockdown of WDR72. HIF-1*α* signal pathway was screened and confirmed as the downstreamWDR72 pathway of WDR72 by dual luciferase assay (Figures S3A and S3B). Thus, we studied whether WDR72 affected the stemness of lung cancer cells relying on the AKT/HIF-1*α* pathway. Western blot results revealed that the abundance of activated AKT was significantly increased and the expression of HIF-1*α* was also significantly up-regulated (Figures 5(a) and 5(b)) after overexpression of WDR72 in A549 stem cells, suggesting that the AKT/HIF-1*α* pathway is involved in WDR72 regulating lung cancer stem cells. Therefore, we treated A549 and SPCA-1 stem cells with PI3K/AKT pathway inhibitor LY29004, and the data demonstrated that LY29004 could counteract the effect of WDR72 overexpression on AKT and HIF-1*α*. At the same time, it can neutralize the effects of WDR72 overexpression on stemness markers, as well as proliferation, metastasis, and apoptosis markers in A549 stem cells. Similar findings were observed in SPCA-1 stem cells (Figures 5(c) and 5(d)). The ability of A549 stem cells to form spheres was found to be enhanced after WDR72 overexpression, but significantly reduced after the addition of LY29004 (Figures S4A and S4B). Furthermore, treatment of cells with LY29004 after overexpression of WDR72 significantly inhibited the proliferation of A549 stem cells (Figures S4E and S4F). In addition, we examined the change in metastatic ability of A549 stem cells, and found that the metastatic ability of LCSCs in the LY29004 treatment group was significantly reduced when compared to the WDR72 overexpression group (Figures S4E and S4F). Besides, the changes in caspase-3 activity also indicated that overexpression of WDR72 could not promote lung cancer stem cell apoptosis after inhibiting the AKT/HIF-1*α* pathway by LY29004 (Figures S4G). These results were further confirmed in SPCA-1 stem cells (Figure S5). Taken together, our findings indicated that WDR72 regulated LCSCs through the AKT/HIF-1*α* signaling pathway.

## 4. Discussion

Lung cancer is the leading cause of cancer-related death among all cancers [32]. Patients with lung cancer have a low survival rate and are prone to recurrence after surgery or radiotherapy treatment, which is currently thought to be due to the presence of CSCs [33]. The CSC theory suggests that although CSCs constitute only a small part of tumor cells, this cell subpopulation has the capacity for carcinogenesis and is the primary reason for cancer development. This theory was supported by a large number of experimental results [34]. Therefore, the strategy of targeting CSCs is the key to overcoming tumor drug resistance. In this study, we investigated the regulatory role of WDR72 on LCSCs and the underlying mechanism.

WDR72 has been sparsely studied in tumors, thus there is a large space for exploration, and our results demonstrate that WDR72 is overexpressed in lung cancer tissues. Similarly, compared with normal cells, the expression of WDR72 was significantly elevated in lung cancer cells, and further elevated in LCSCs, suggesting that WDR72 is involved in the regulation of lung cancer cell stemness. We then knocked down WDR72 in LCSCs, and the results showed that inhibition of WDR72 markedly decreased the sphere-forming ability of LCSCs and the abundance of stemness-related genes such as OTC4, CD44, and CD133. The impact of knocking down WDR72 on the function of LCSCs was evaluated. The findings revealed that knockdown of WDR72 restrained the growth and metastasis of LCSCs and promoted apoptosis. In addition, in vivo experiments further demonstrated that knockdown of WDR72 deterred tumorigenesis of LCSCs. Overall, our results demonstrate that WDR72 is overexpressed in lung cancer tissues and LCSCs, and that inhibition of WDR72 deters the stemness and protumorigenic effects of LCSCs. To investigate the mechanism by which WDR72 affects LCSCs, we detected the expression of key molecules of tumor-associated signaling pathways.

The PI3K/AKT pathway is the critical pathways in cells [35]. Aberrant activation of the PI3K/AKT pathway enhances characteristics of tumors such as increased proliferation, cell cycle progression, metastasis, and inhibition of apoptosis [36, 37]. In lung cancer, the PI3K/Akt signaling pathway is frequently overactivated [38, 39]. The PI3K/AKT pathway also participates in the regulation of LCSCs [35]. A study revealed that FBLN3 restrained the stemness of lung cancer cells via the IGF1R/PI3K/AKT/GSK3*β* signaling pathway [40]. PI3K is activated when cells are stimulated by external stimuli and subsequently, PI3K catalyzes the production of PIP3, which binds to AKT and phosphorylates it [41]. Activation of AKT is often considered a hallmark of cancer, promoting tumor cell proliferation and migration, inhibiting tumor cell apoptosis, increasing tumor mutation rates, and thus promoting cancer progression [42]. HIF-1*α* is an important hypoxic stress factor induced by tumor hypoxic microenvironment and is closely related to several aspects of tumor formation. HIF-1*α* is downstream of the PI3K/AKT pathway, and the activation of AKT has an important impact on the function of HIF-1*α*. A study showed that overexpression of miR-204 could inhibit the metastasis of NSCLC cells by suppressing the AKT/HIF-1*α* axis [43]. The findings in this study illustrated that WDR72 upregulation significantly activated the AKT/HIF-1*α* pathway, and the abundance of p-AKT and HIF-1*α* was markedly elevated. Application of PI3K/AKT pathway inhibitor LY29004 was able to counteract the influence of WDR72 upregulation on stemness, metastasis, apoptosis, and proliferative gene expression in lung cancer stem cells. After inhibiting the AKT/HIF-1*α* pathway, the sphere-forming ability of LCSCs was significantly reduced. The promotion of WDR72 overexpression on lung cancer stem cell proliferation and metastasis was also eliminated by LY29004 treatment. Taken together, WDR72 can regulate the AKT/HIF-1*α* signaling pathway to enhance the stemness of LCSCs and promote the growth and metastasis of lung cancer, but its application in clinical diagnosis and treatment needs to be researched further.

## 5. Conclusions

The study demonstrated that WDR72 is overexpressed in lung cancer, particularly in LCSCs. Knocking down of WDR72 was efficient to suppress the growth and metastasis of LCSCs and deterred tumor growth in BALB/c nude mice via regulating the AKT/HIF-1*α* signaling pathway. These findings indicate that WDR72 could be a potential treatment target for lung cancer.

## Data Availability

The original data presented in this study are included in the article/Supplementary Material, and further inquiries can be directed to the corresponding authors.

## Supplementary Materials

Supplementary Materials Figure S1: Immunohistochemistry (IHC) staining examines WDR72 expression in adjacent tissues and cancer tissues, and the staining density was determined using Image *J* software. Figure S2: The public database “Kaplan-Meier Plotter” (https://kmplot.com/analysis/index.php?p=service) shows high expression of WDR72 in NSCLC tumors correlate poor clinical outcome. Figure S3: (A) The Cignal Finder Cancer 10-Pathway Reporter Array screened WDR72-related signal pathways in CSC cells. (B) 3 × HRE luciferase assay system confirmed HIF-1*α* signal pathway is downregulated when knockdown WDR72 in NSCLC CSC cells. Figure S4: WDR72 regulates A549 stem cell proliferation, migration, and apoptosis via the AKT/HIF-1*α* pathway. (A) Evaluation of tumor sphere formation after treatment with AKT inhibitor. (B) Statistical data revealed the size of the spheres in the dedicated groups. (C) The proliferative ability of A549 stem cells measured by colony formation. (D) The changes in colony number after LY29004 treatment. (E) The migration of A549 stem cells measured by Transwell assay. (F) The number of migrated cells. (G) The abundance of caspase-3 measured to evaluate the change in apoptotic ability. Figure S5: WDR72 regulates SPCA-1 stem cells proliferation, migration, and apoptosis via the AKT/HIF-1*α* signaling pathway. (A) Evaluation of tumor sphere formation after treatment with AKT pathway inhibitor. (B) Statistical data revealed the size of the spheres in the dedicated groups. (C) The proliferative ability of SPCA-1 stem cells measured by colony formation. (D)The changes in colony number after LY29004 treatment. (E) The migration of SPCA-1 stem cells measured by Transwell assay. (F) The number of migrated cells. (G) The abundance of caspase-3 measured to evaluate the change in apoptotic ability.
