# Supplementary figures and images for: WDR72 Enhances the Stemness of Lung Cancer Cells by Activating the AKT/HIF-1α Signaling Pathway
Source: J Oncol. 2022 Nov 7;2022:5059588. doi: 10.1155/2022/5059588 (PMC9663245; doi:10.1155/2022/5059588)

**A**

## IHC: anti-WDR72

Adjacent tissue

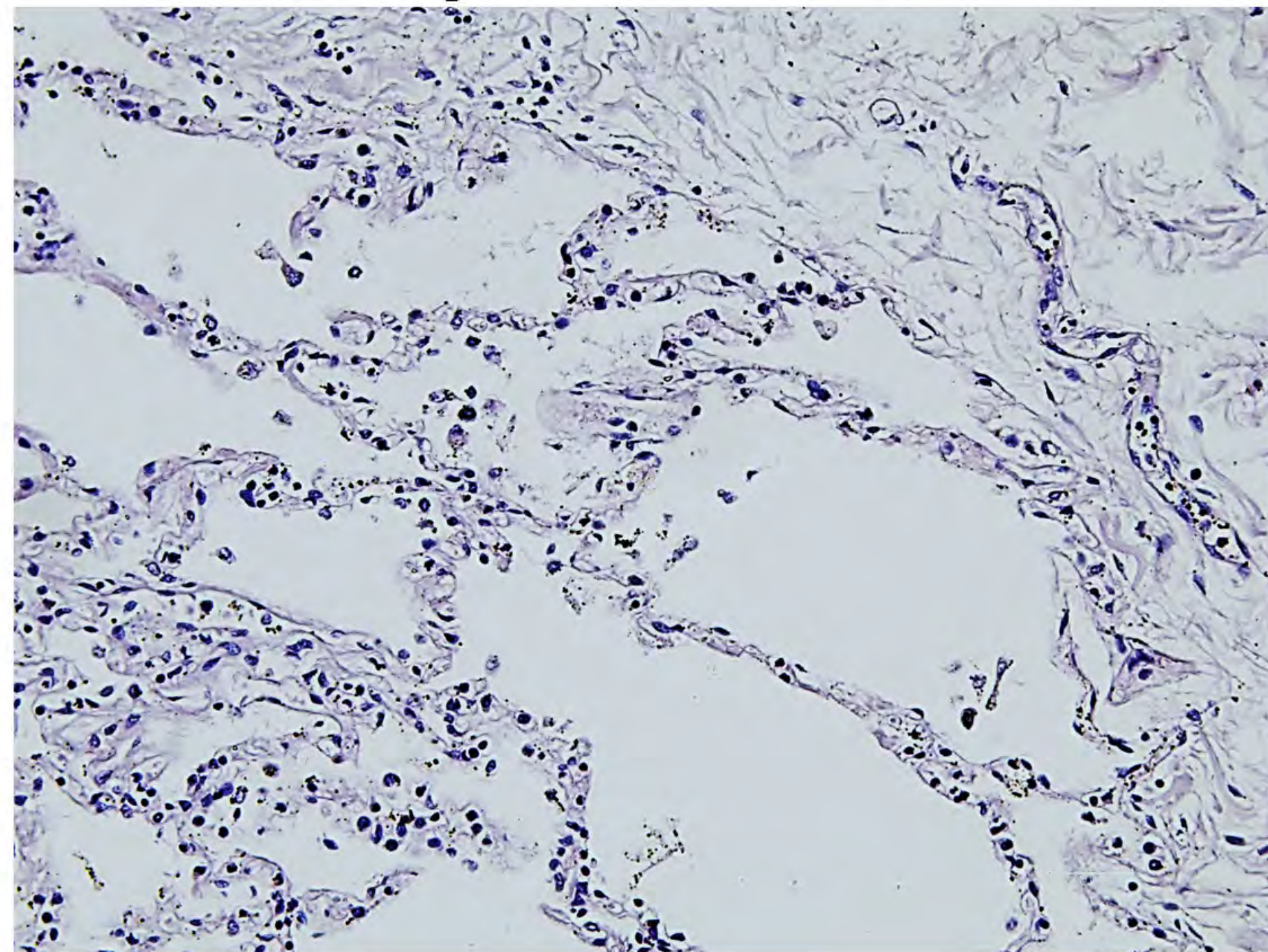

Cancer tissue

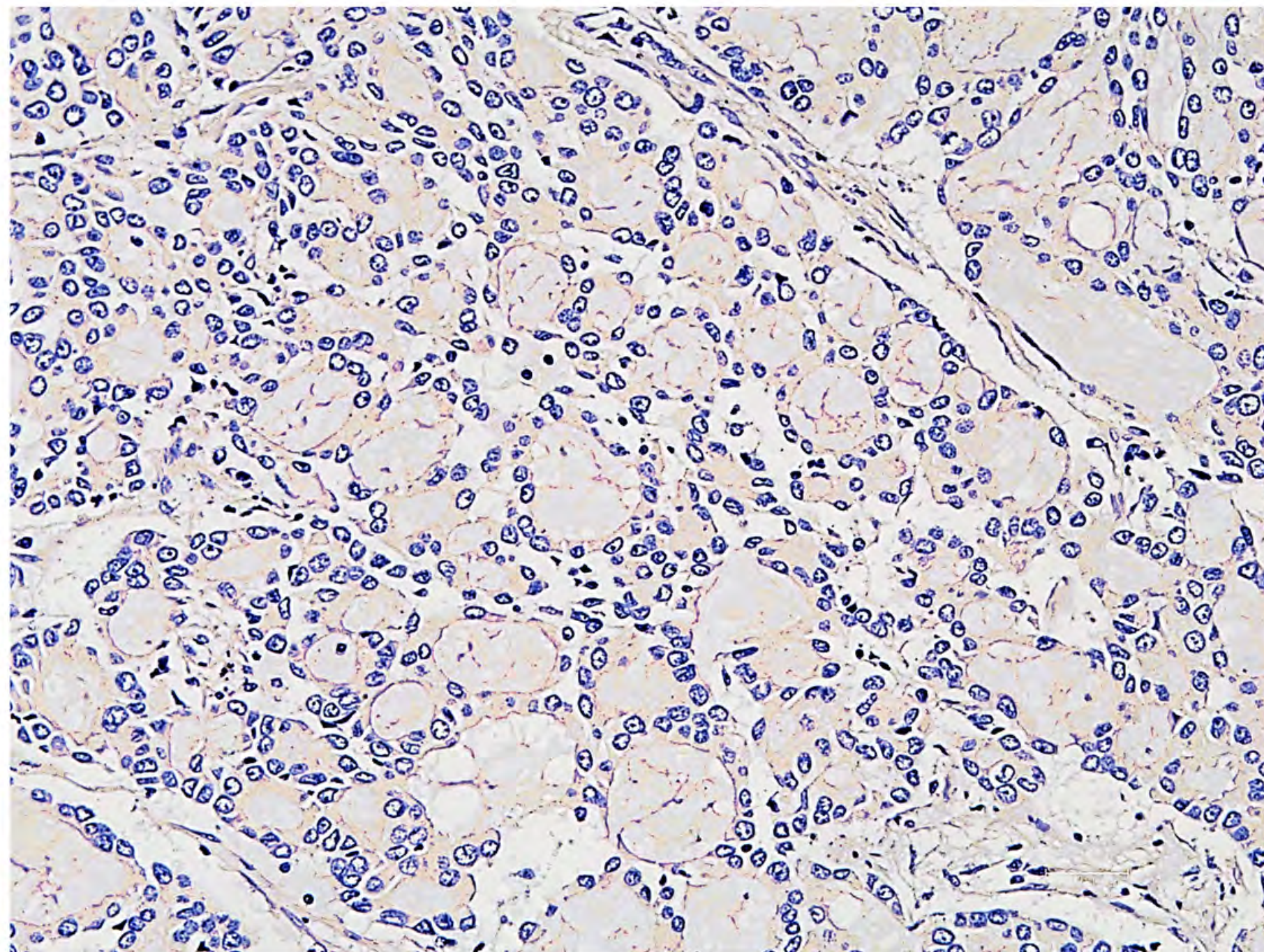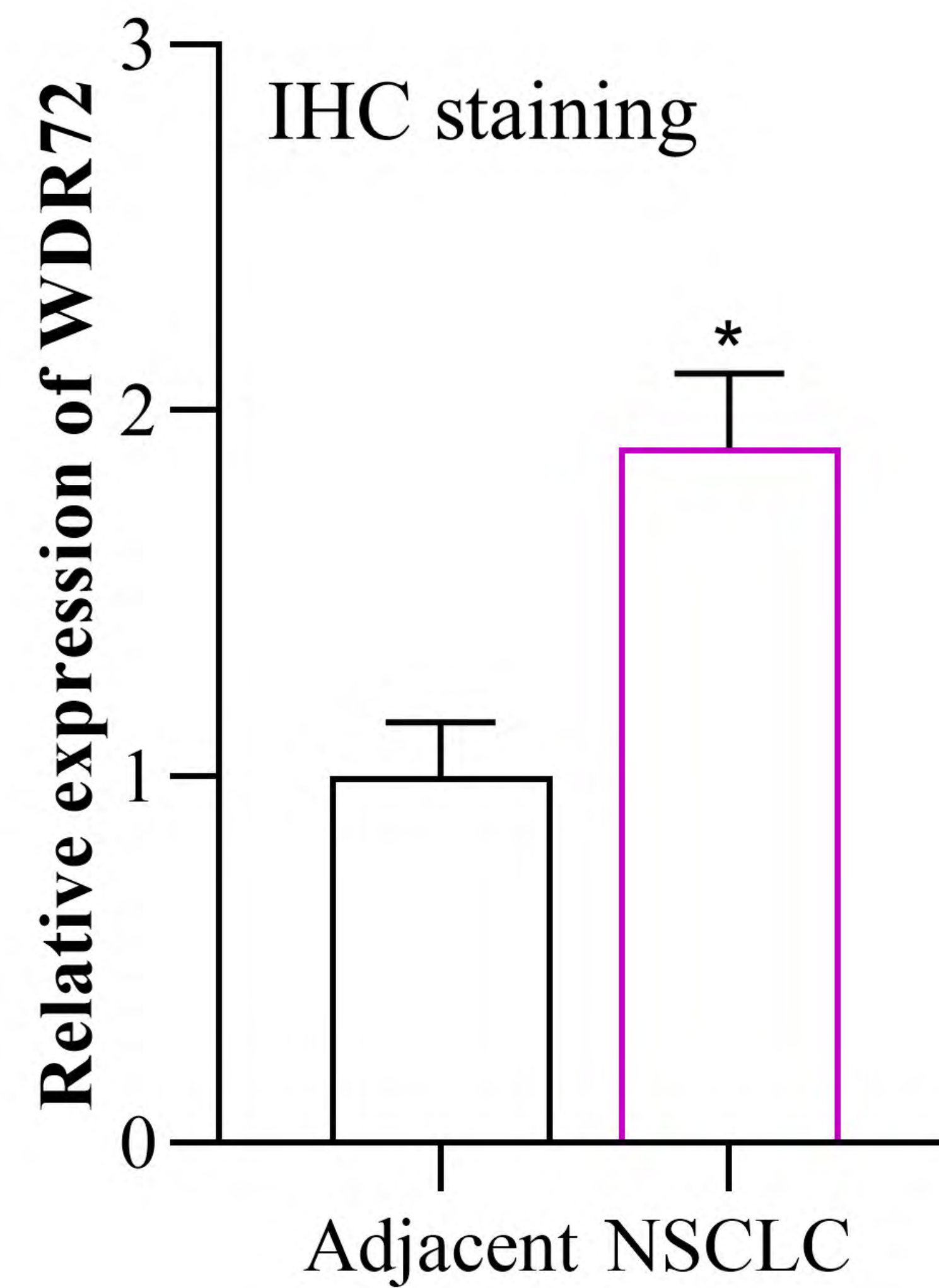

**A****WDR72 (227174\_at)**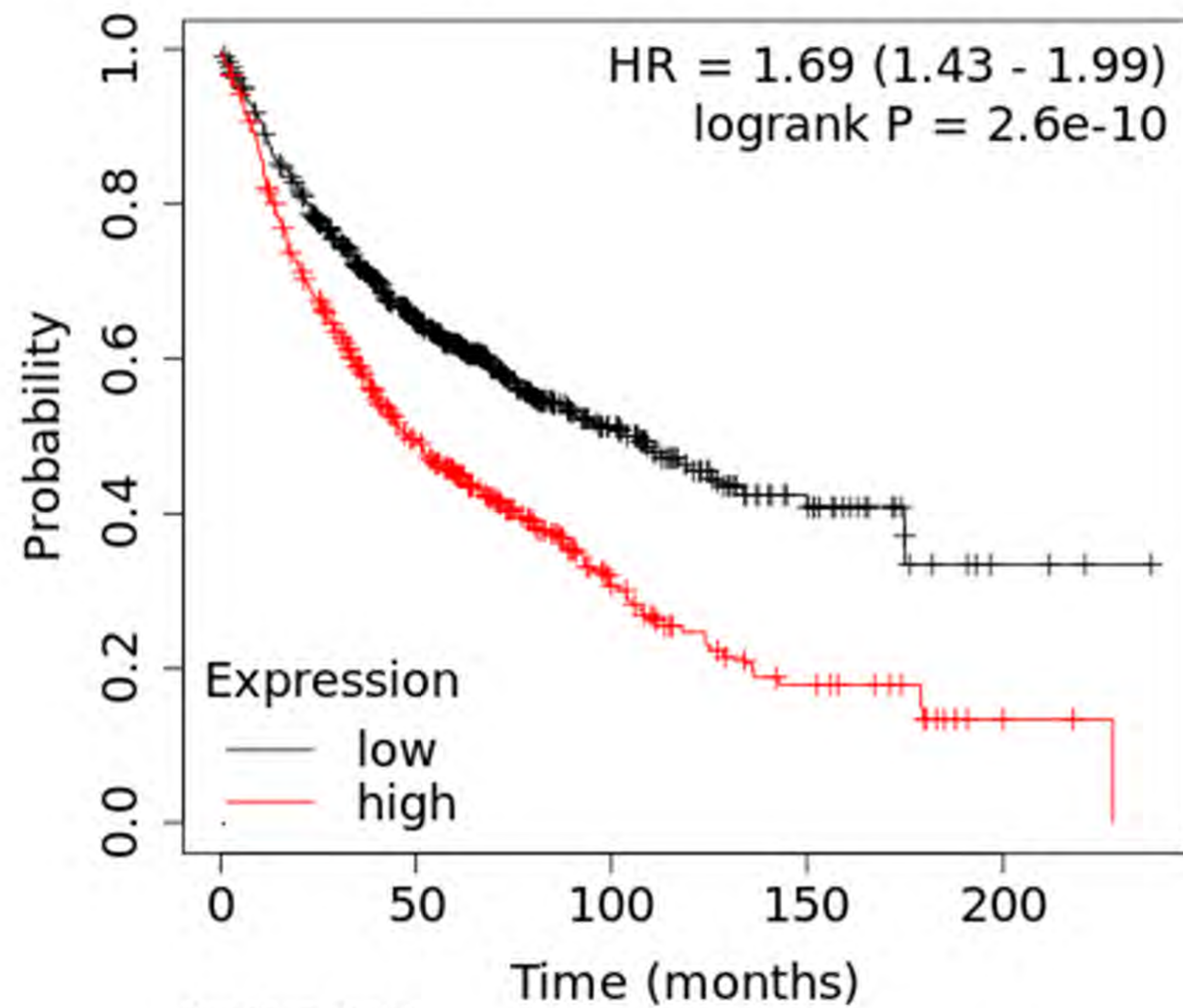

| Number at risk |     |     |    |    |   |  |
|----------------|-----|-----|----|----|---|--|
| low            | 725 | 373 | 89 | 27 | 3 |  |
| high           | 419 | 172 | 50 | 19 | 3 |  |

**A**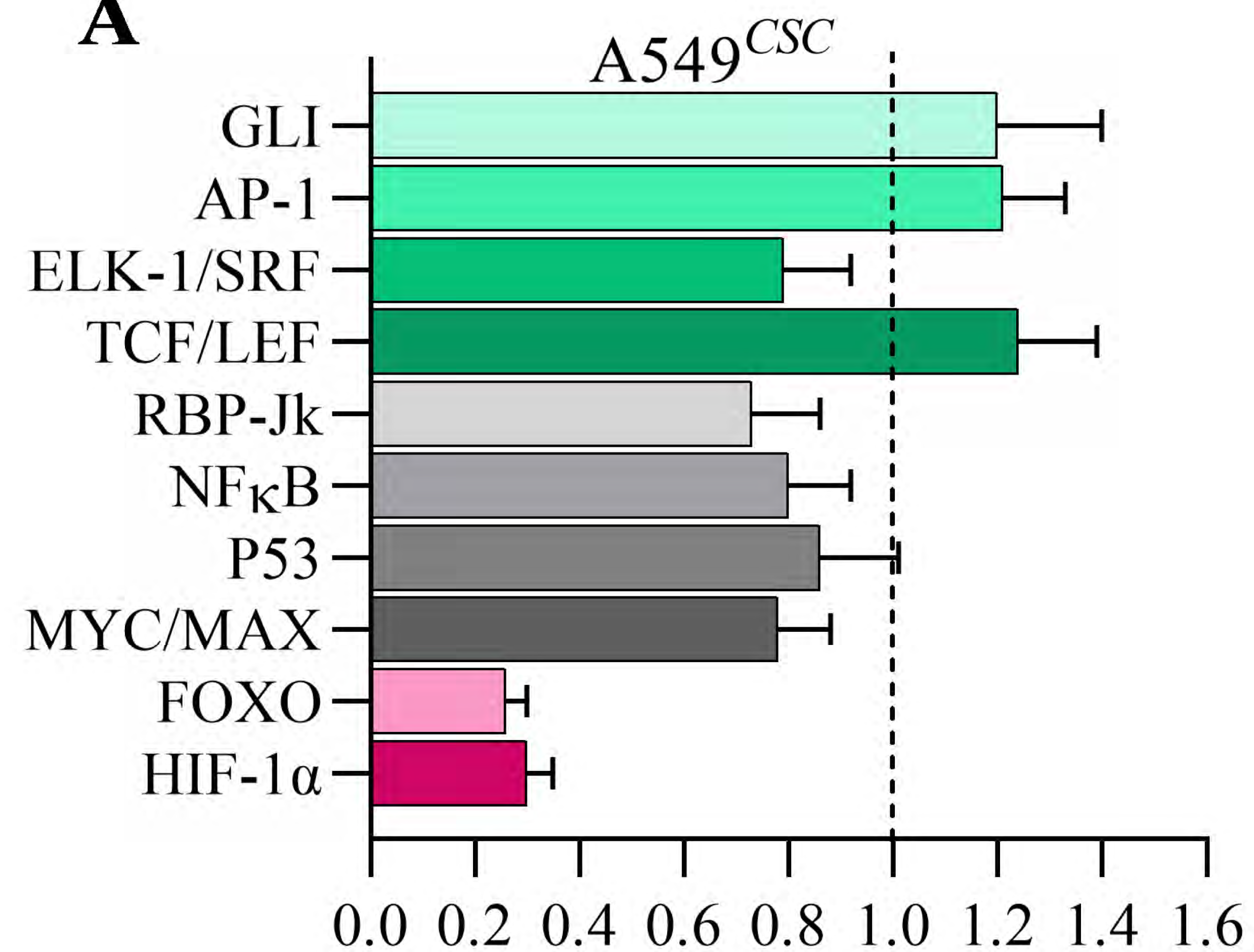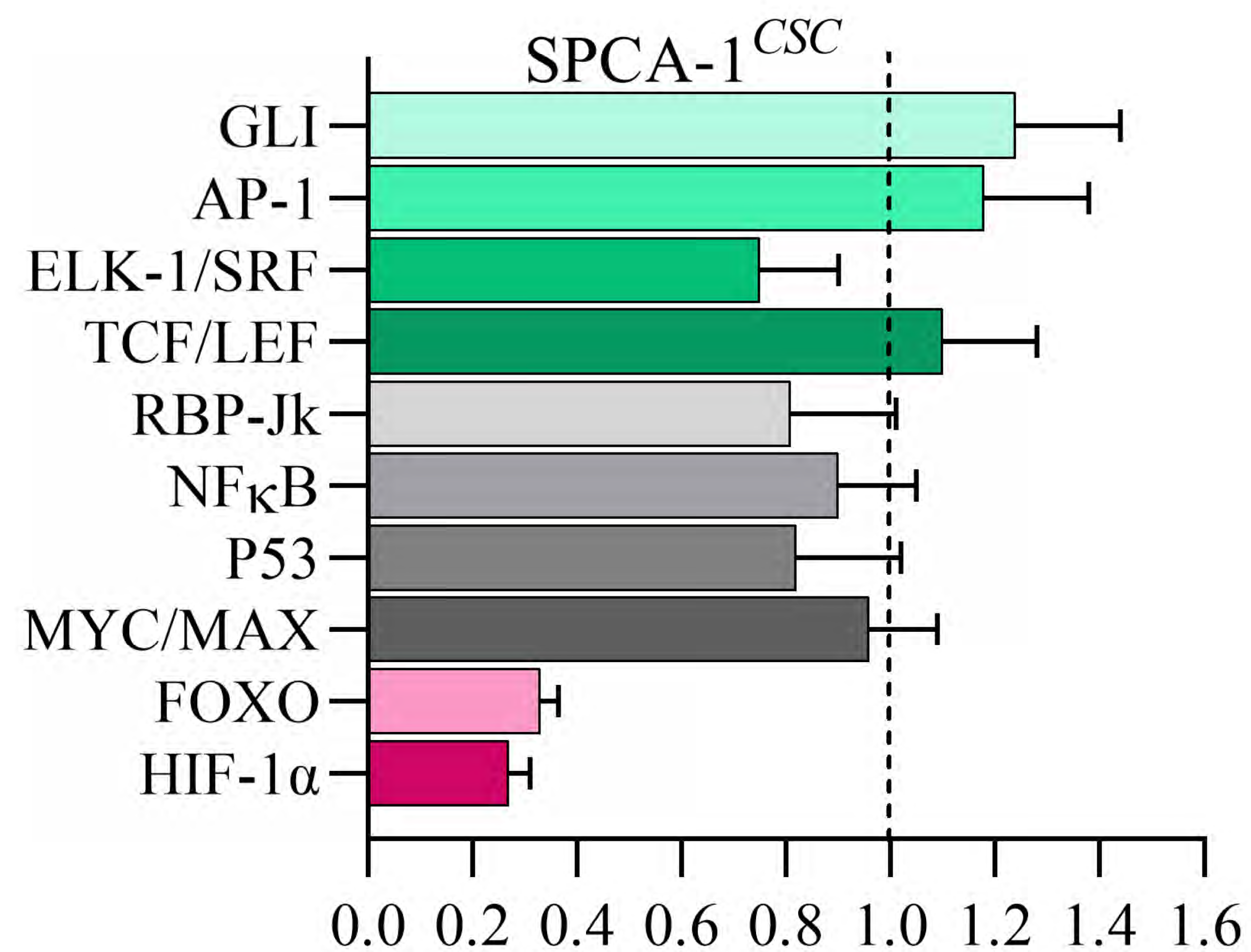**B**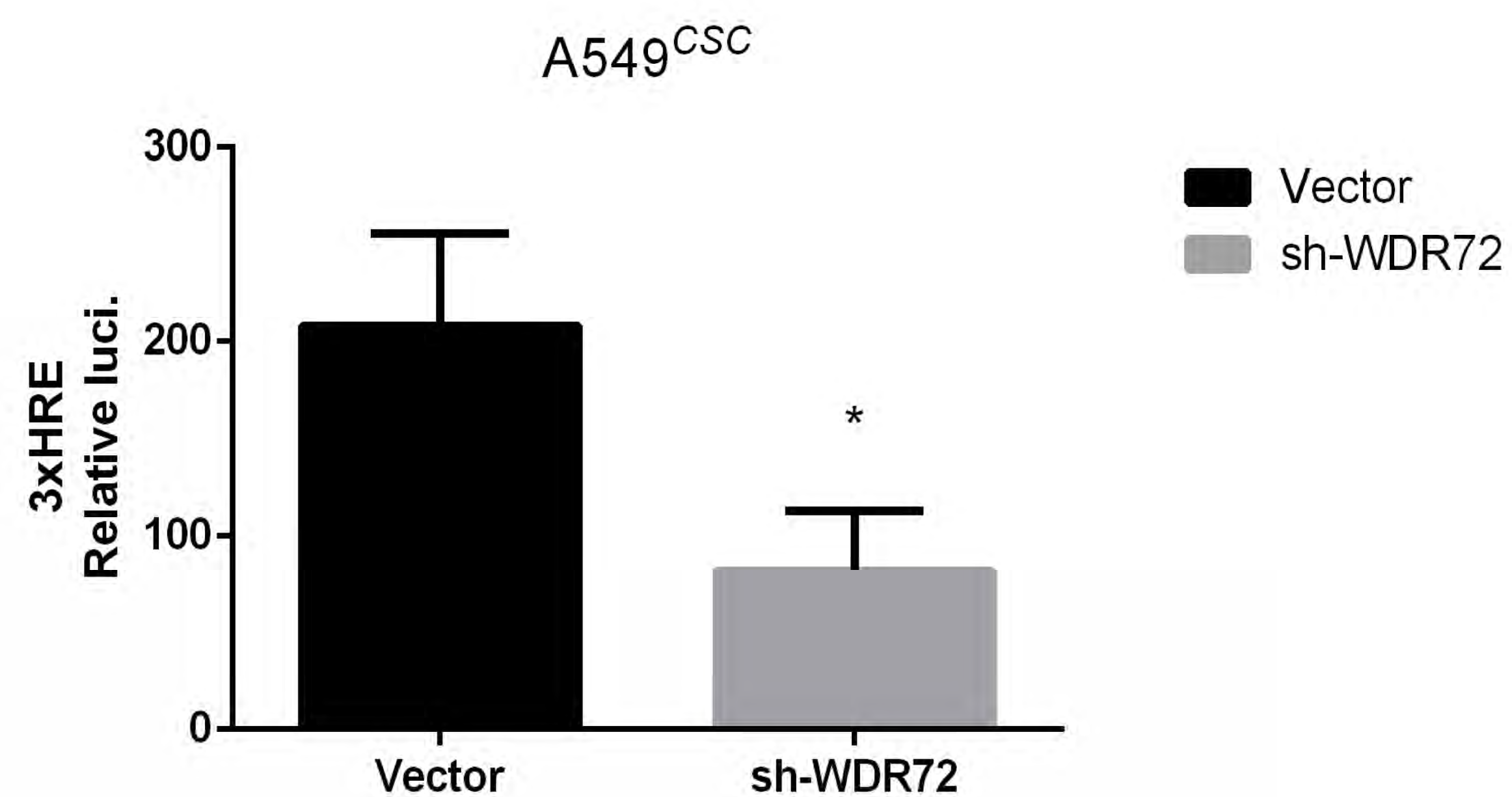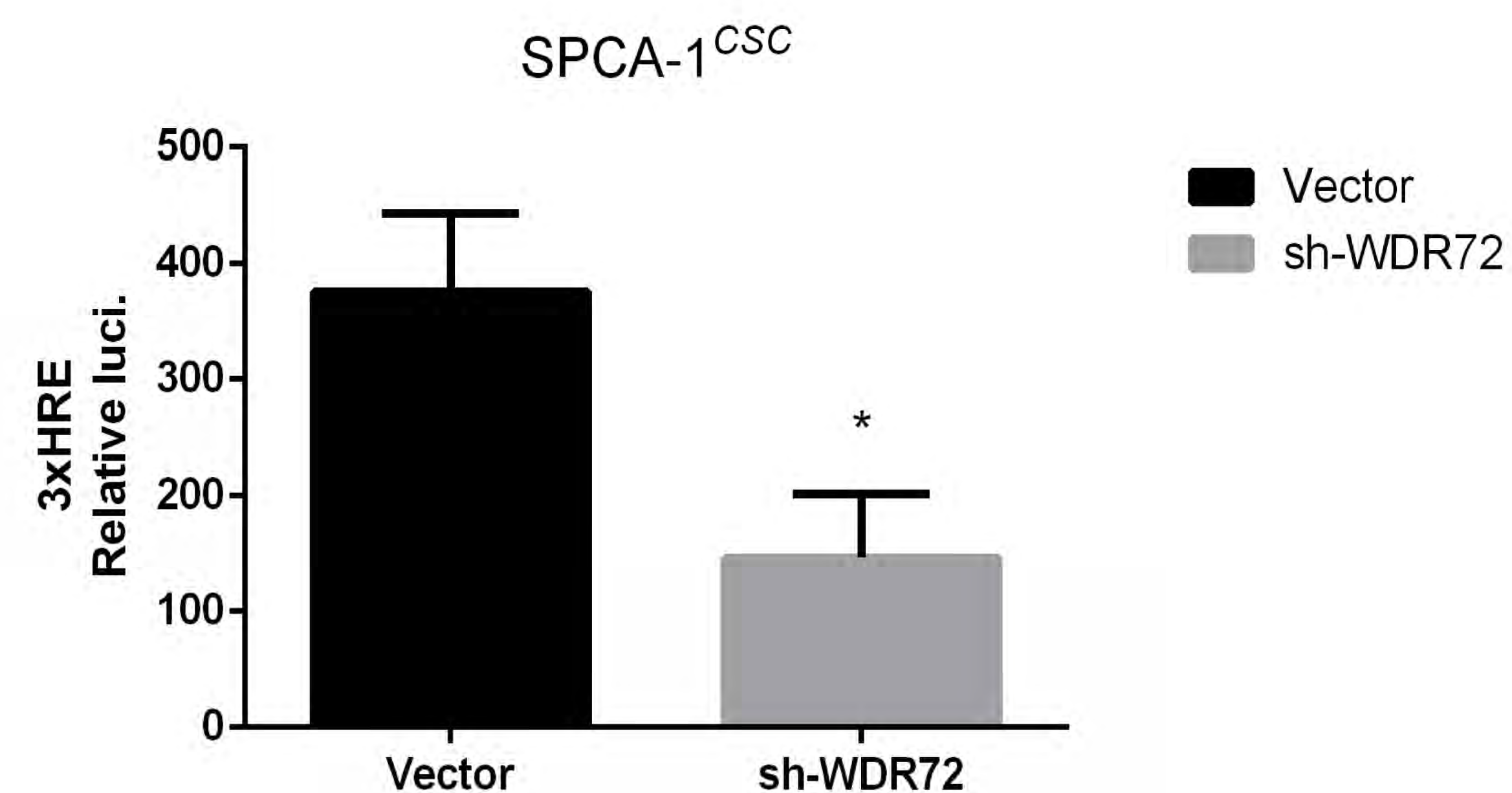

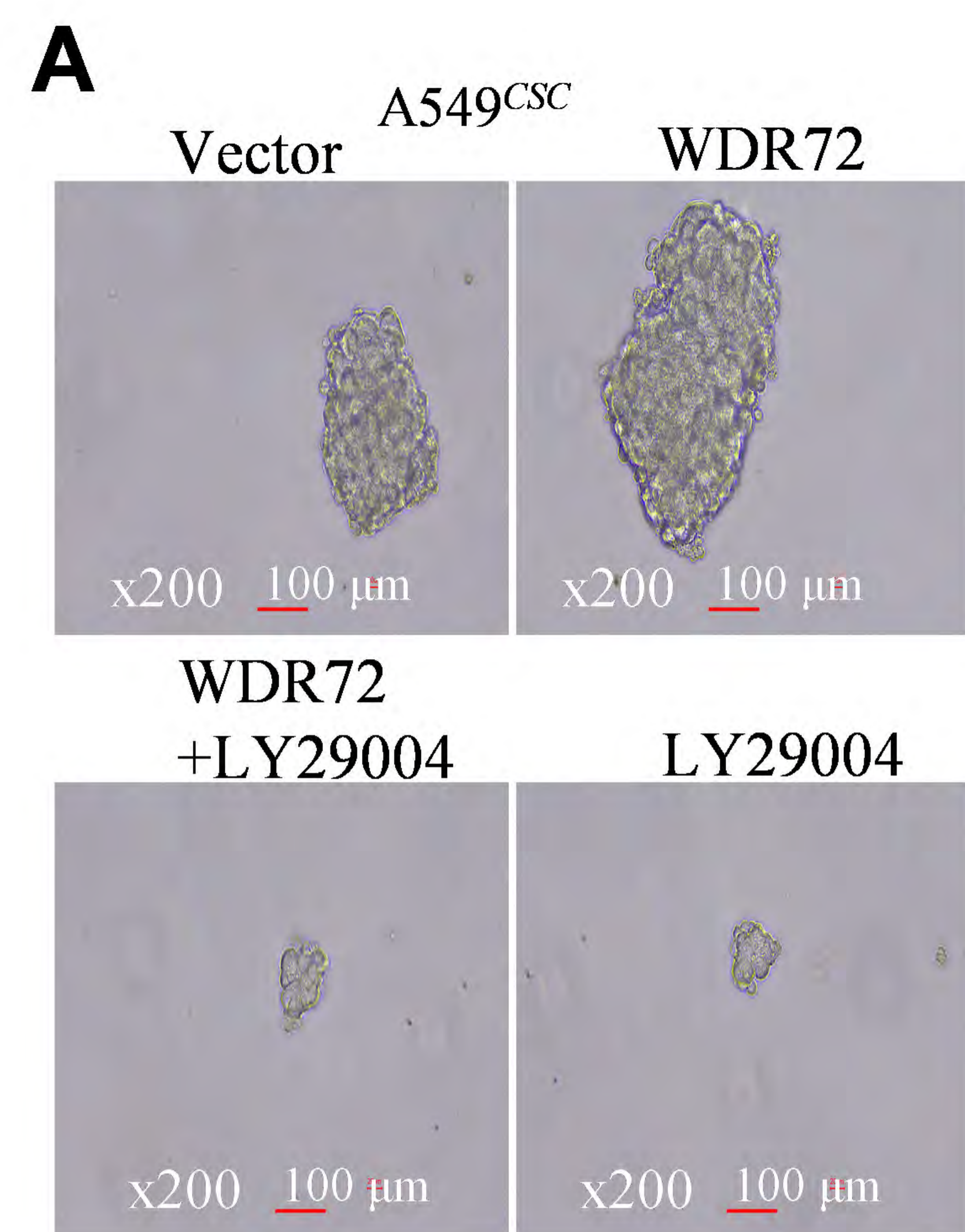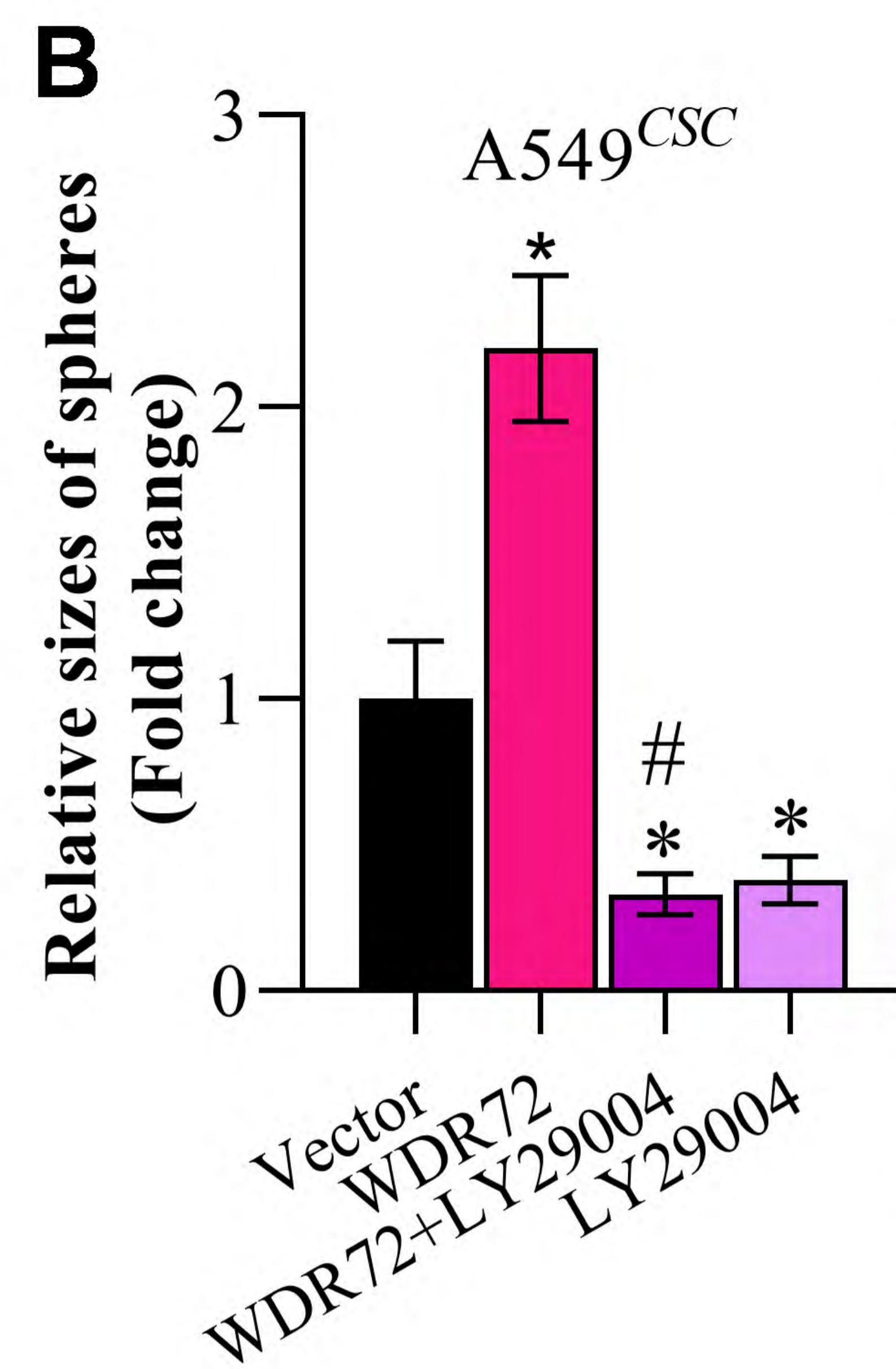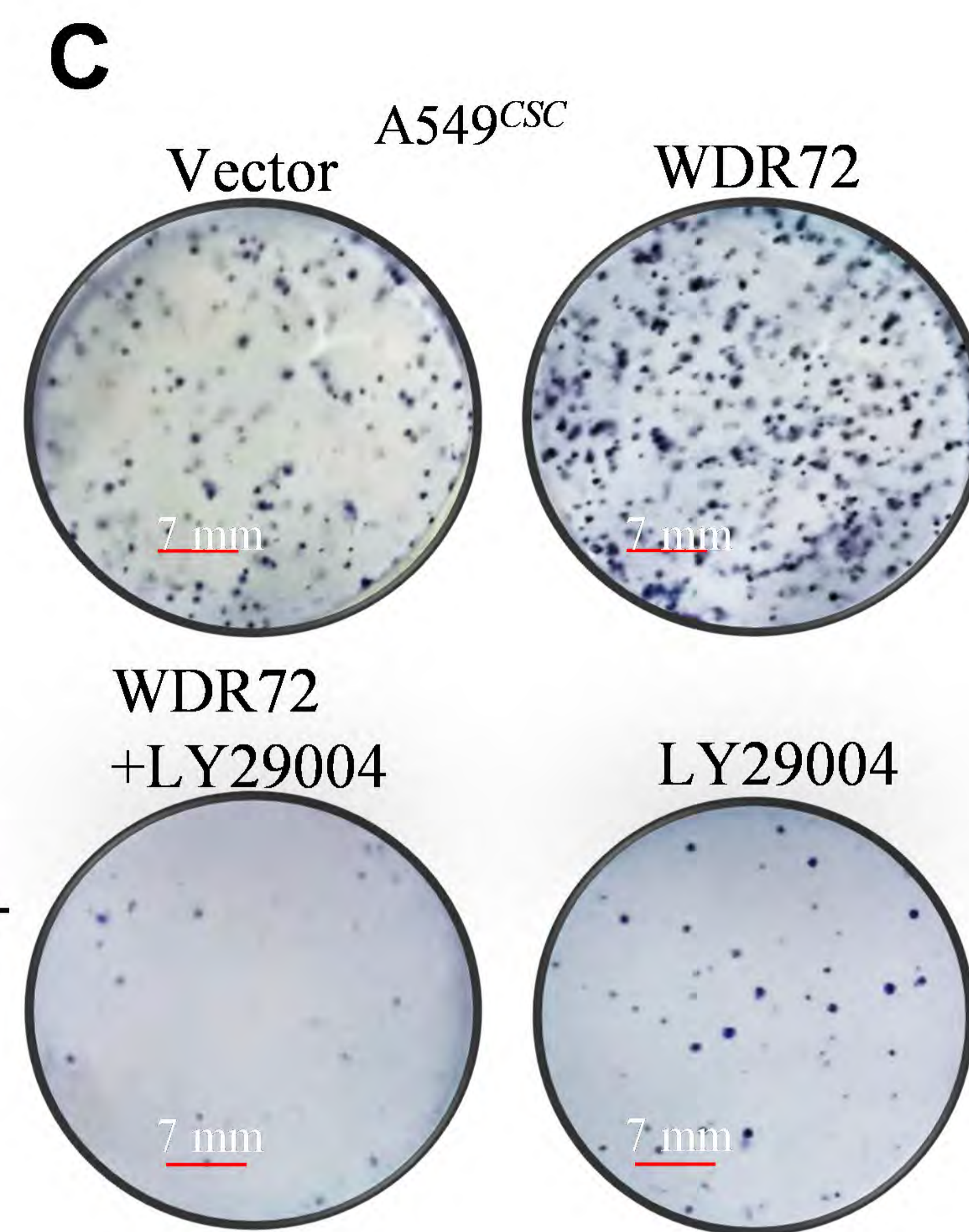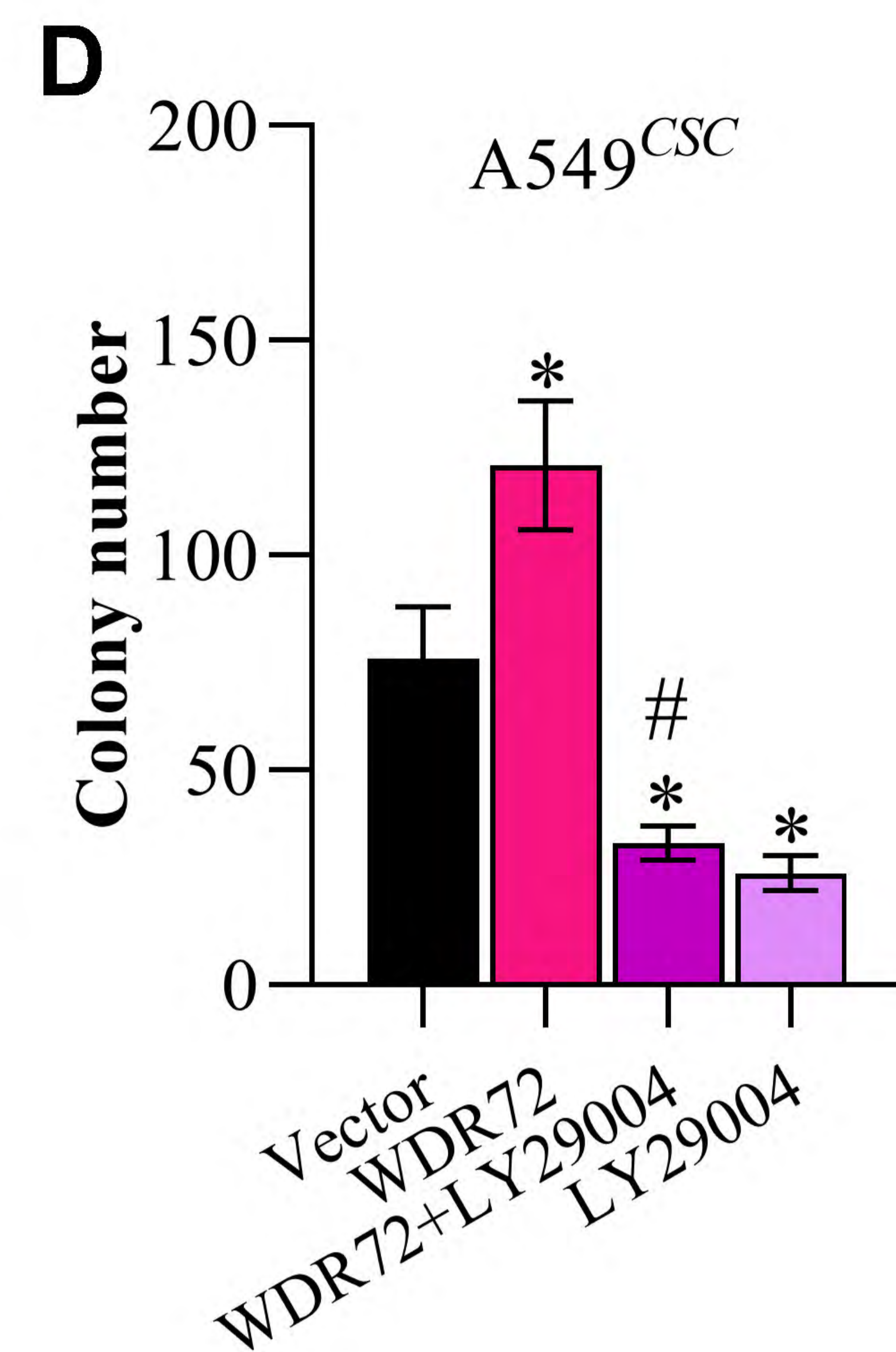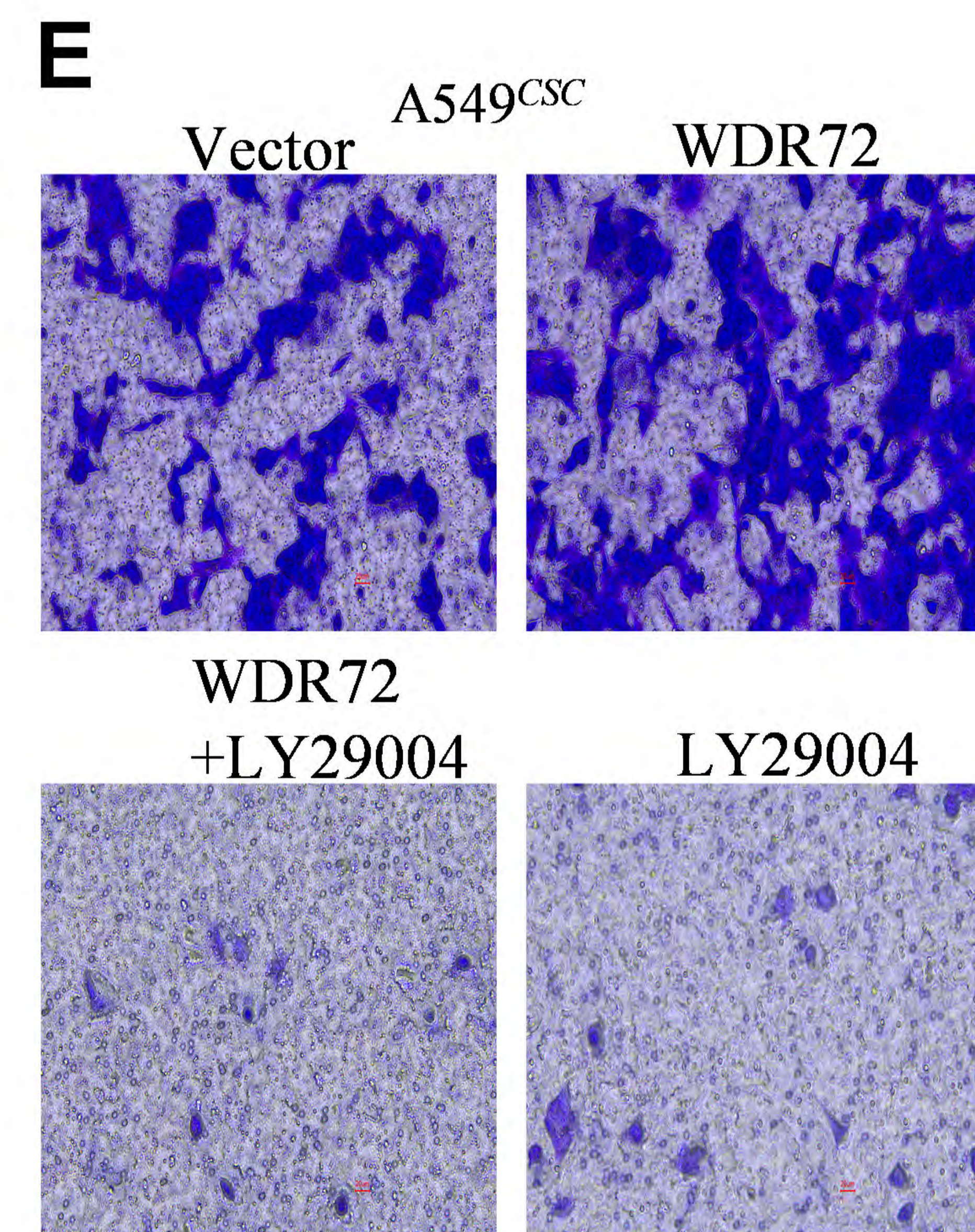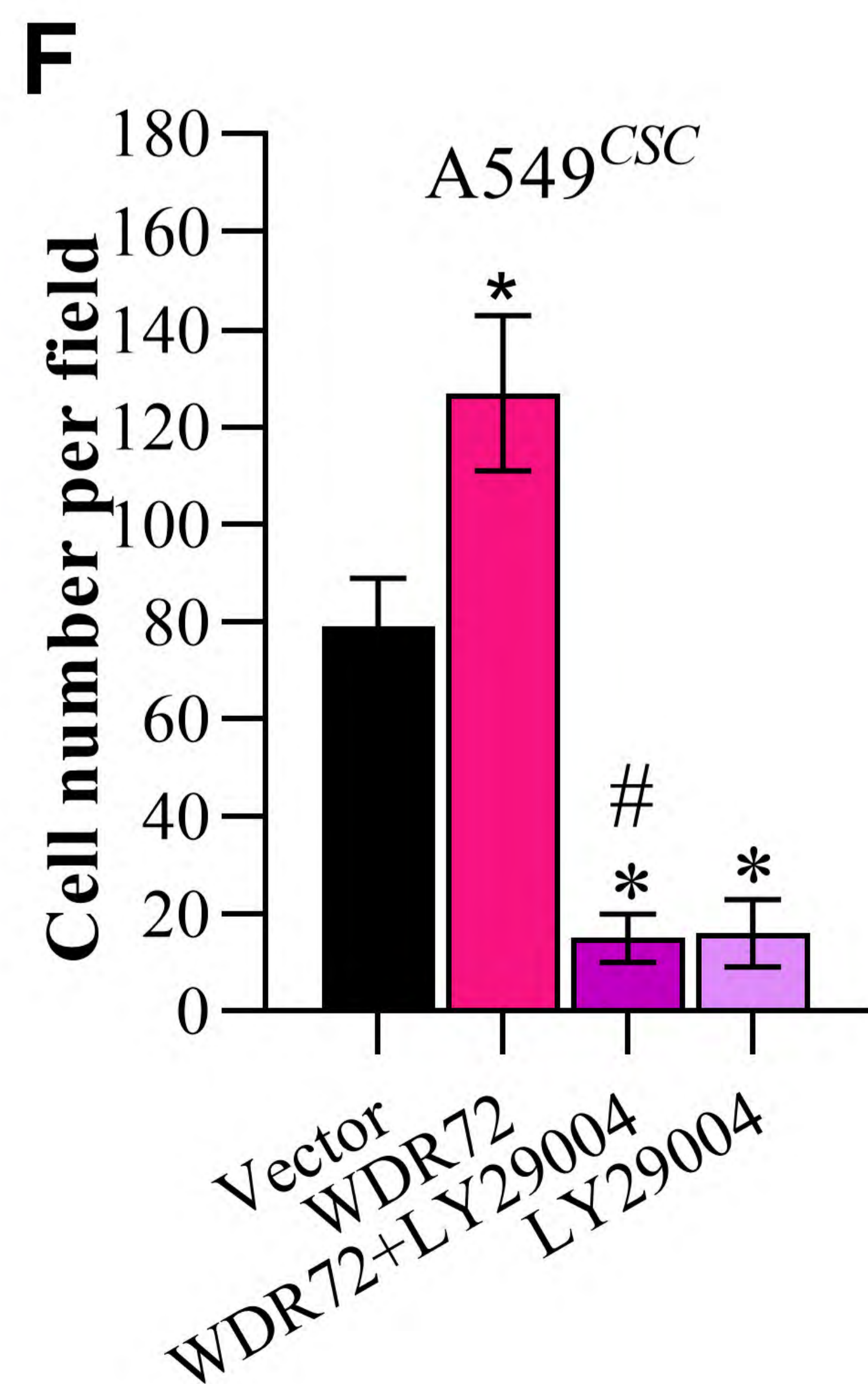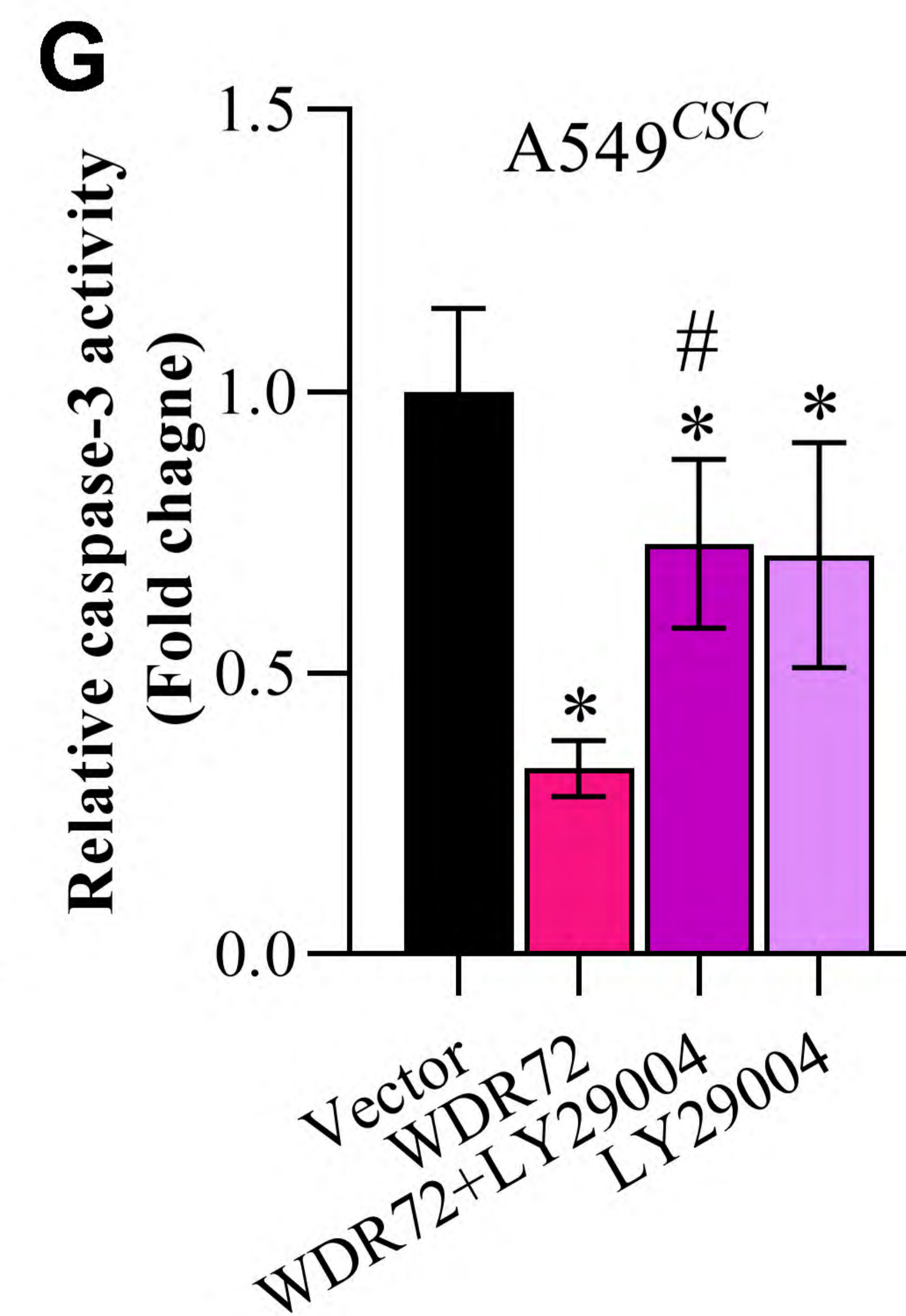

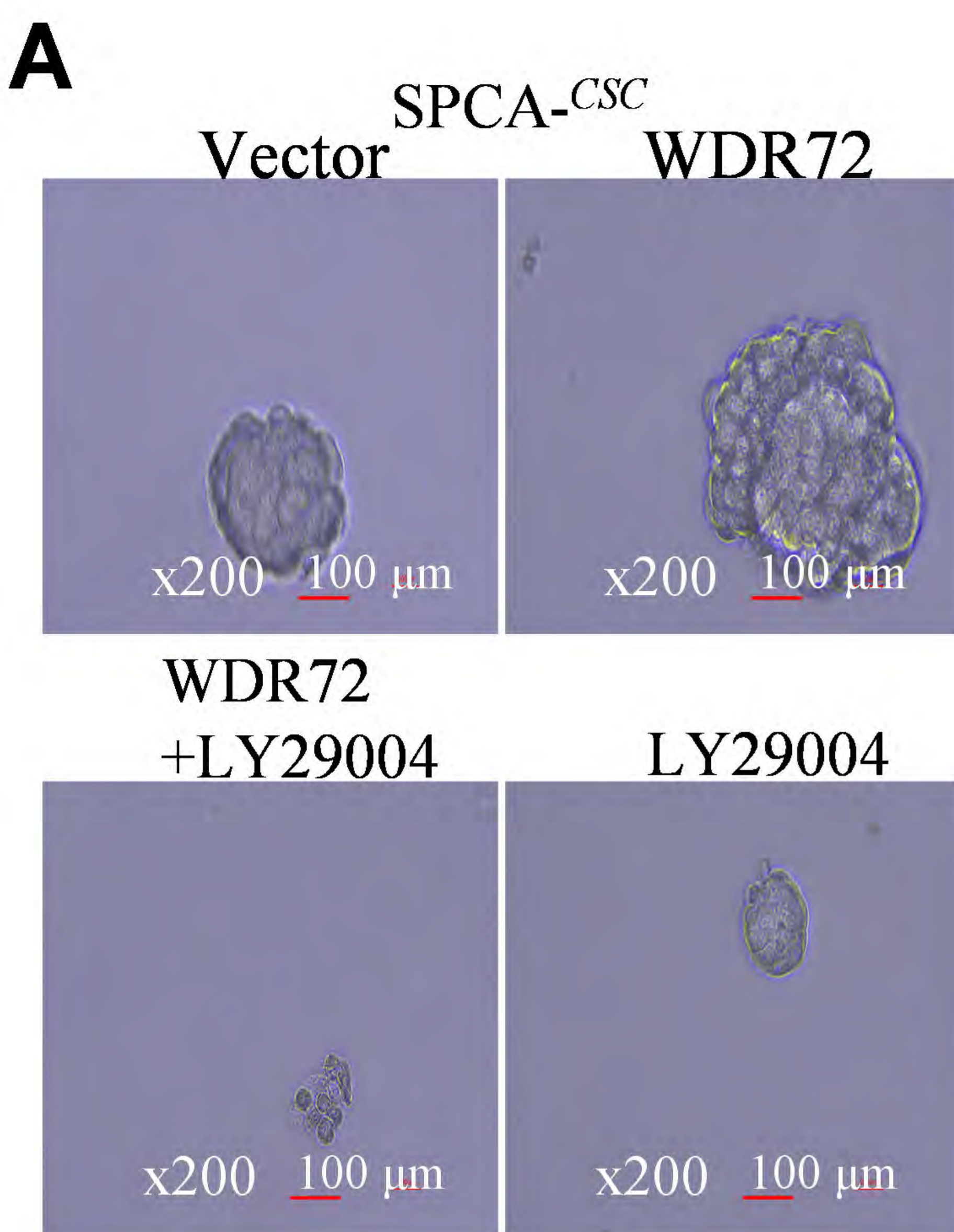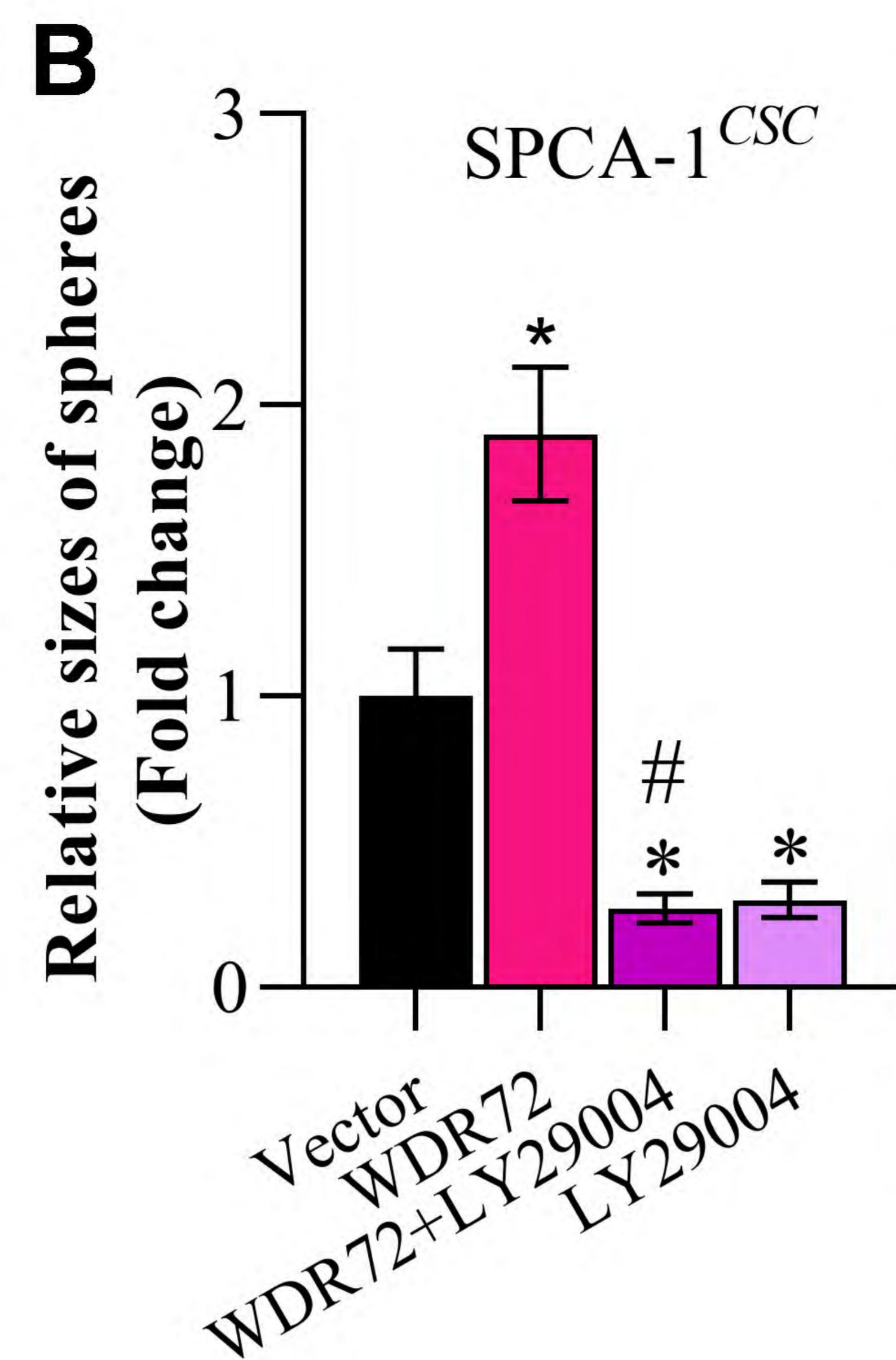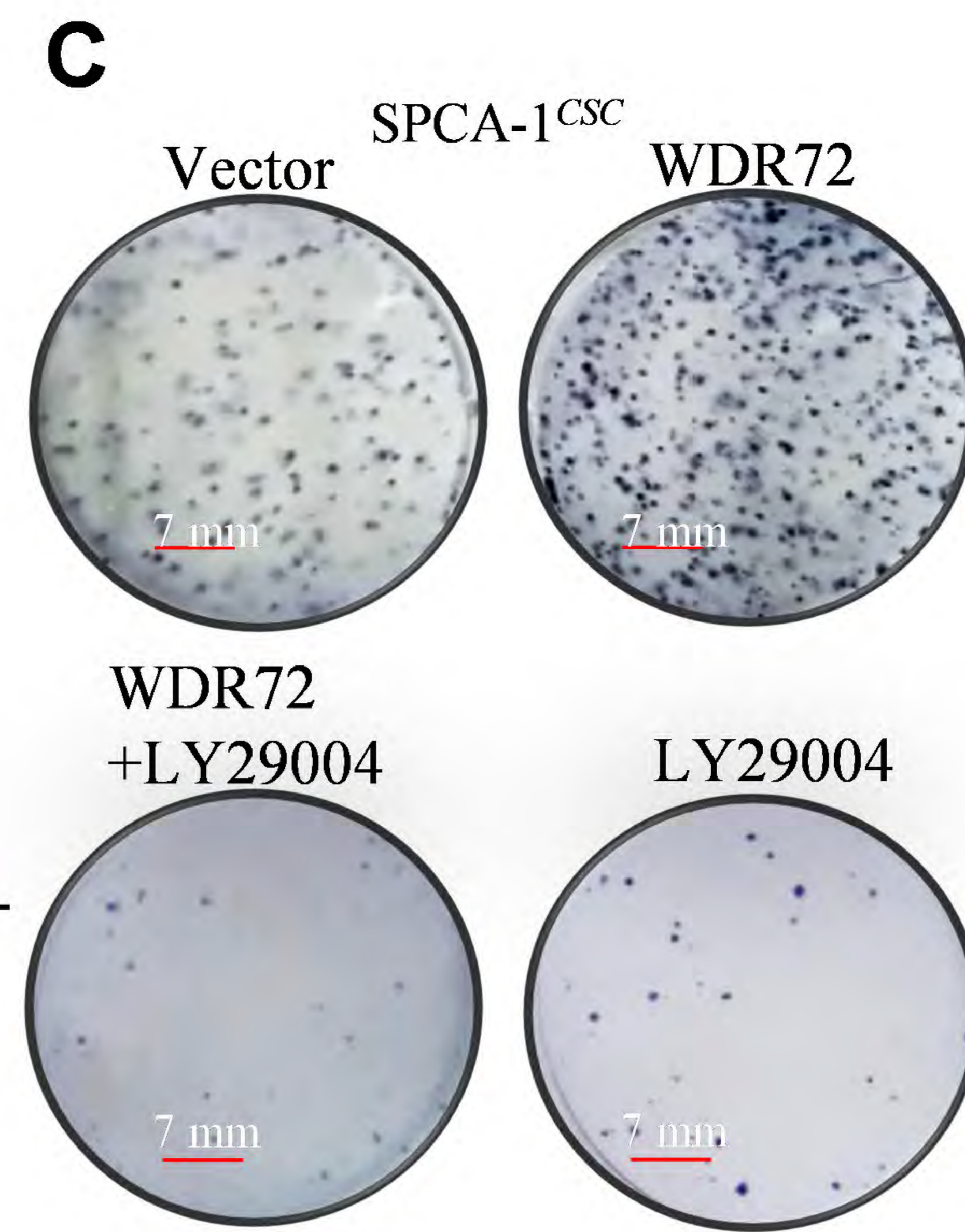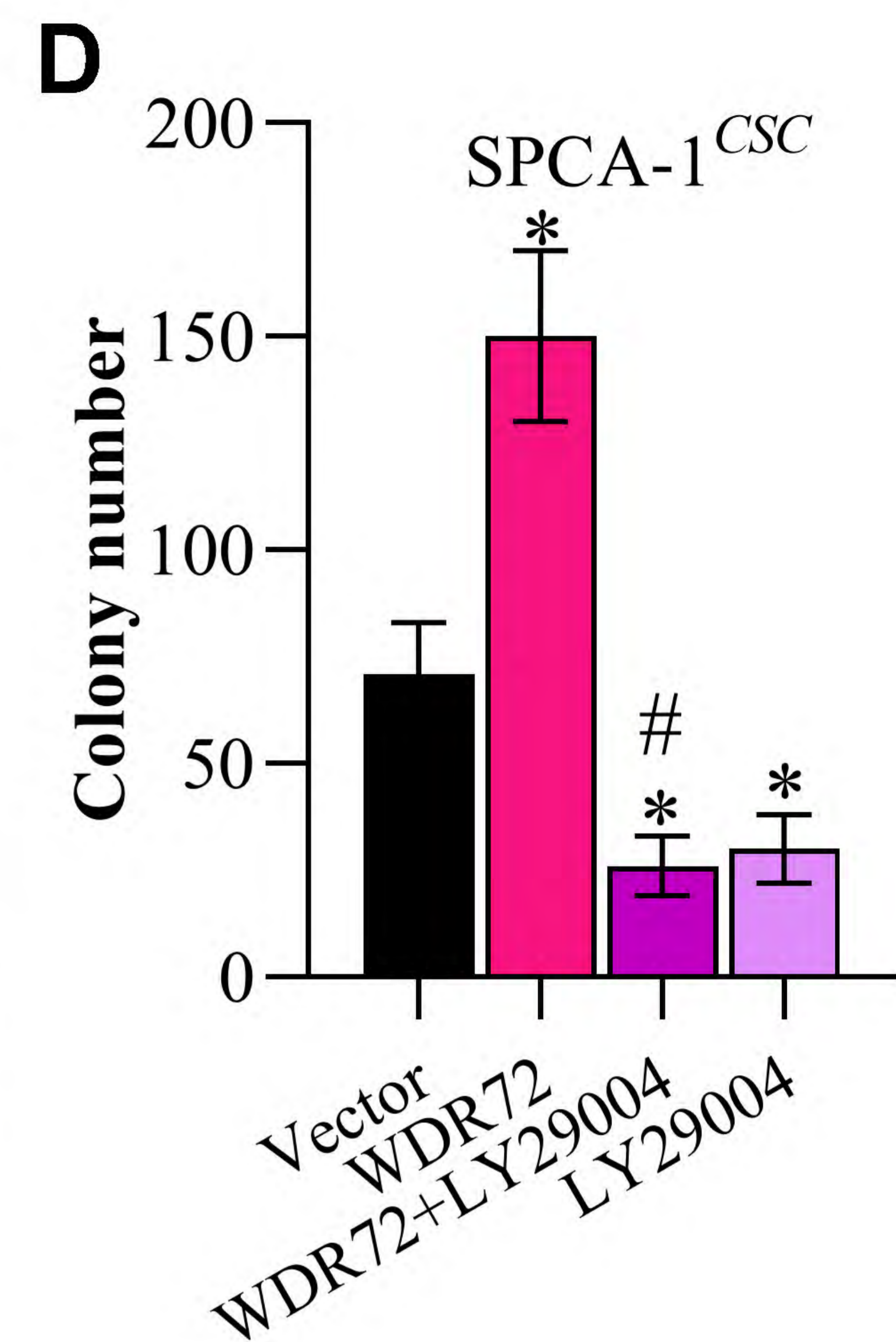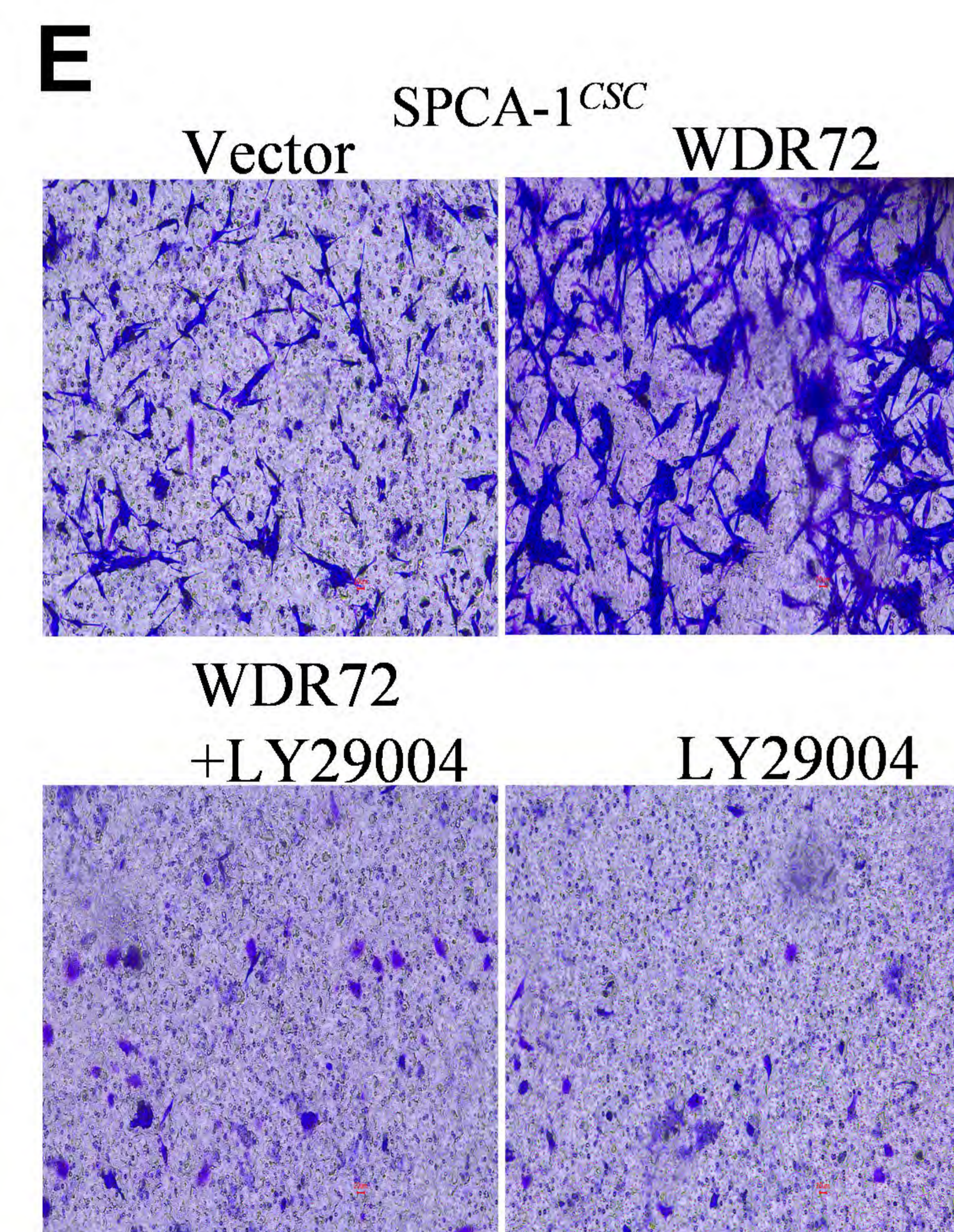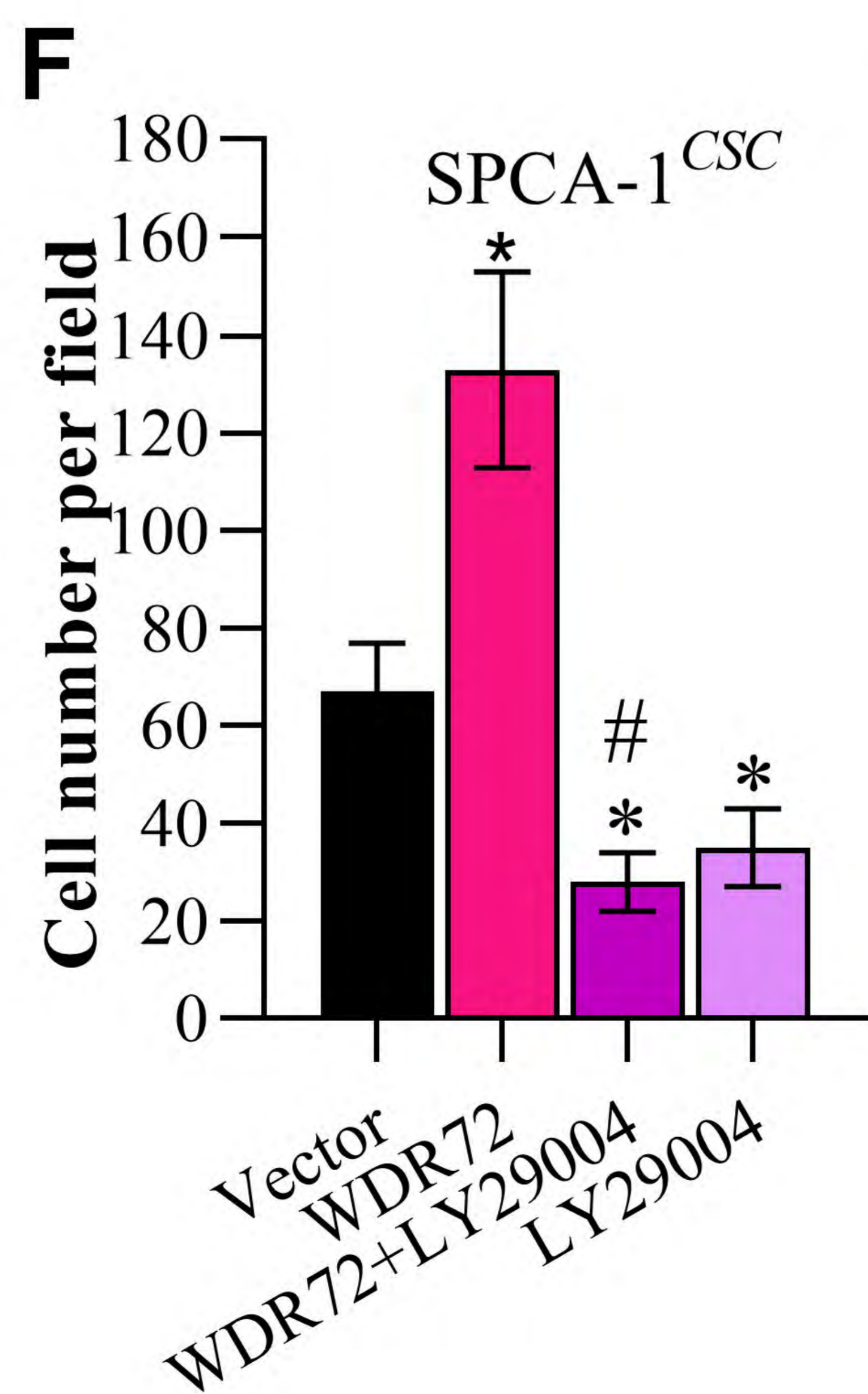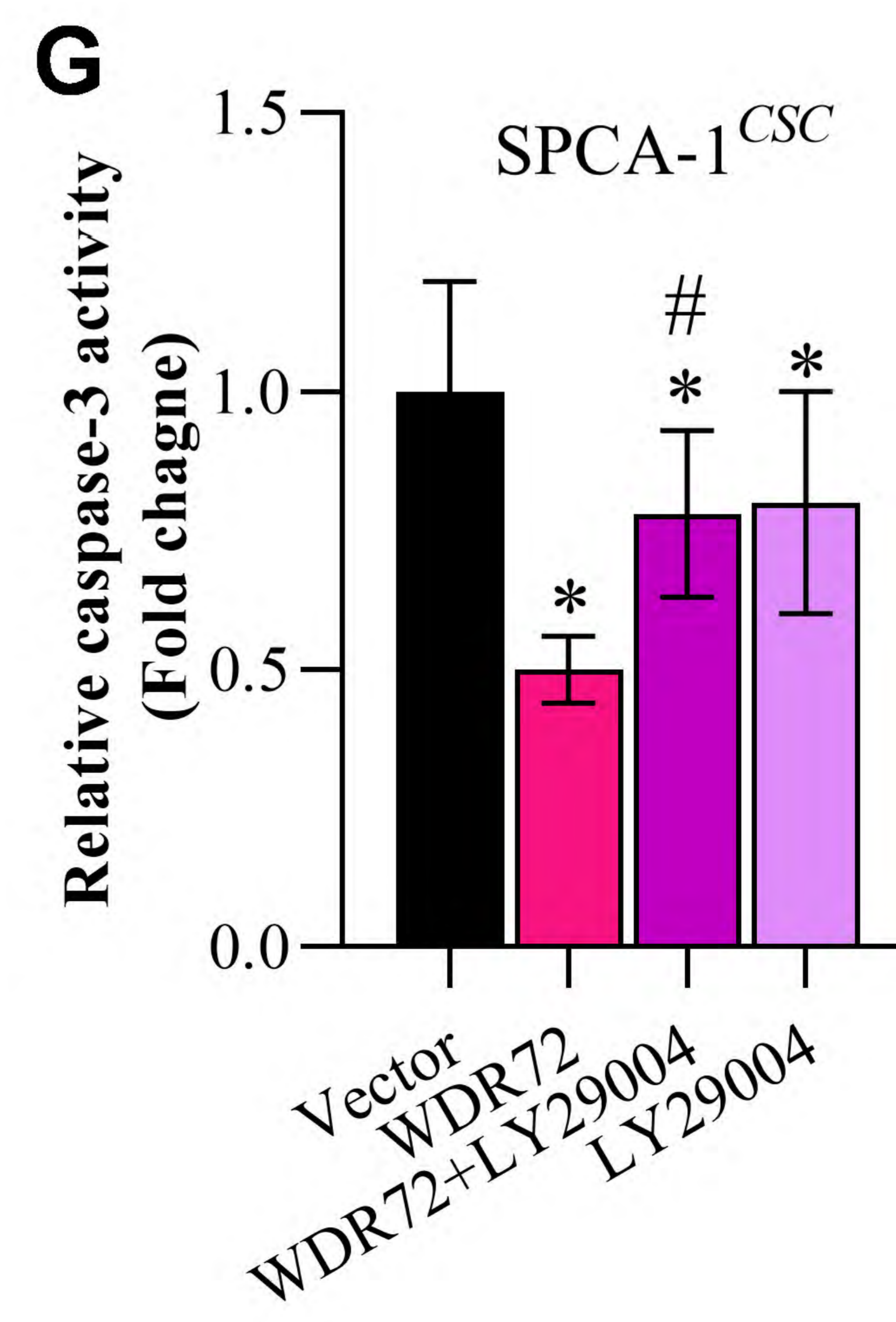

Supplement: Supplementary Materials — Figure S1: Immunohistochemistry (IHC) staining examines WDR72 expression in adjacent tissues and cancer tissues, and the staining density was determined using Image J software. Figure S2: The public database “Kaplan-Meier Plotter” (https://kmplot.com/analysis/index.php?p=service) shows high expression of WDR72 in NSCLC tumors correlate poor clinical outcome. Figure S3: (A) The Cignal Finder Cancer 10-Pathway Reporter Array screened WDR72-related signal pathways in CSC cells. (B) 3 × HRE luciferase assay system confirmed HIF-1α signal pathway is downregulated when knockdown WDR72 in NSCLC CSC cells. Figure S4: WDR72 regulates A549 stem cell proliferation, migration, and apoptosis via the AKT/HIF-1α pathway. (A) Evaluation of tumor sphere formation after treatment with AKT inhibitor. (B) Statistical data revealed the size of the spheres in the dedicated groups. (C) The proliferative ability of A549 stem cells measured by colony formation. (D) The changes in colony number after LY29004 treatment. (E) The migration of A549 stem cells measured by Transwell assay. (F) The number of migrated cells. (G) The abundance of caspase-3 measured to evaluate the change in apoptotic ability. Figure S5: WDR72 regulates SPCA-1 stem cells proliferation, migration, and apoptosis via the AKT/HIF-1α signaling pathway. (A) Evaluation of tumor sphere formation after treatment with AKT pathway inhibitor. (B) Statistical data revealed the size of the spheres in the dedicated groups. (C) The proliferative ability of SPCA-1 stem cells measured by colony formation. (D)The changes in colony number after LY29004 treatment. (E) The migration of SPCA-1 stem cells measured by Transwell assay. (F) The number of migrated cells. (G) The abundance of caspase-3 measured to evaluate the change in apoptotic ability. [file 5059588.f1.pdf]
